# Transcriptome Profiling During Muscadine Berry Development Reveals the Dynamic of Polyphenols Metabolism

**DOI:** 10.3389/fpls.2021.818071

**Published:** 2022-02-02

**Authors:** Ahmed Ismail, Ahmed G. Darwish, Minkyu Park, Pranavkumar Gajjar, Violeta Tsolova, Karam F. A. Soliman, Islam El-Sharkawy

**Affiliations:** ^1^Center for Viticulture and Small Fruit Research, College of Agriculture and Food Sciences, Florida A&M University, Tallahassee, FL, United States; ^2^Department of Horticulture, Faculty of Agriculture, Damanhour University, Damanhour, Egypt; ^3^Department of Biochemistry, Faculty of Agriculture, Minia University, Minia, Egypt; ^4^College of Pharmacy and Pharmaceutical Sciences, Institute of Public Health, Florida A&M University, Tallahassee, FL, United States

**Keywords:** antioxidant activity, berry development, flavonoid, muscadine grape, phenolic, transcriptome profiling

## Abstract

Muscadine grapes accumulate higher amounts of bioactive phenolics compared with other grape species. To identify the molecular events associated with polyphenolic accumulation that influence antioxidant capacity, two contrasting muscadine genotypes (C5 and C6) with varied phenolic/flavonoid content and antioxidant activity were investigated *via* RNA-sequencing during berry development. The results showed that berry development is concomitant with transcriptome profile changes, which was more pronounced at the véraison (V) stage. Despite that the downregulation pattern of gene expression dominated the upregulation through berry development, the C5 genotype maintained higher expression levels. Comparative transcript profiling allowed the identification of 94 differentially expressed genes with potential relevance in regulating fruit secondary metabolism, including 18 transcription factors and 76 structural genes. The genes underlying the critical enzymes in the modification reactions of polyphenolics biosynthetic pathway, including hydroxylation, methylation, and glycosylation were more pronounced during the immature stages of prevéraison (PrV), V, and postvéraison (PoV) in the C5 genotype, resulting in more accumulation of biologically active phenolic/flavonoid derivatives. The results suggested that muscadine grapes, as in bunch grapes (*Vitis* sp.); possess a similar mechanism that organizes polyphenolics accumulation; however, the set of total flavonoids (TFs) and structural genes coordinating the pathway varies between the two species.

## Introduction

Grapevine is a member of the large genus *Vitis* that is divided into two subgenera with a varying number of somatic chromosomes (Olien, 1990). The *Euvitis* (bunch grapes) displays a large group that is classified based on the geographical origin into three groups, including the European (*V. vinifera*), the East Asian (*V. amurensis*), and the North American species (*V. riparia*, *V. labrusca*, etc.) (Šikuten et al., 2020). However, *Muscadinia* is a smaller group that comprises three species, including *M. rotundifolia*, *M. munsoniana*, and *M. popenoei* (Olien, 1990). The muscadine grapes (*M. rotundifolia*), unlike other *Muscadinia* species, are commercially valuable growing mainly for fresh fruit and wine production but with lesser popularity than the European grapes (Andersen et al., 2021). However, muscadines are receiving growing attention due to their tolerance to several diseases that cause extensive economic losses in bunch grapes (Staudt, 1997; Merdinoglu et al., 2003). Additionally, muscadine berries display enhanced nutraceutical value due to the accumulation of distinctive phytochemical constituents that have a great potential activity against several chronic diseases (Bralley et al., 2007; God et al., 2007; Mellen et al., 2010; Gourineni et al., 2012; Luo et al., 2017; Mendonca et al., 2019). Such nutritional and health merits are not only restricted to muscadine, but muscadine berries accumulate higher amounts of bioactive polyphenolics compared with other grape species (Pastrana-Bonilla et al., 2003; You et al., 2012; Mendonca et al., 2019).

Polyphenolic compounds are a large group of plants’ secondary metabolites synthesized in multiple sequential pathways, including the pentose phosphate, shikimate, and phenylpropanoid pathways (Randhir et al., 2004). They have substantial functions in plants, including growth, development, and defense. Polyphenolics also define berry quality and sensory attributes, such as texture, flavor, and pigmentation properties (Gomez-Plaza et al., 2001; Duan et al., 2019). The composition and amount of phenolics are highly coordinated by many factors, including genotypic variations, environmental conditions, and more importantly, developmental stages (Darwish et al., 2021). Grape berry development exhibits a double-sigmoid growth pattern, including (I) berry formation, (II) lag phase, and finally (III) ripening phase (Coombe and McCarthy, 2000). Stages I and III are well-known for their exponential increase in berry size, a missing feature in the lag phase. During immature stages (I and II), the accumulation of organic acids, mainly malic and tartaric acids, raises berry acidity levels (Sweetman et al., 2012). However, the acidity declines when the critical point known as véraison occurs at the end of stage II. Furthermore, the berry begins accumulating sugars (mainly glucose and fructose) and anthocyanins in colored varieties. These physiological and biochemical changes proceed during the ripening stage that exhibits a striking reduction in the organic acid coincided with a considerable accumulation of sugar and pigments (Dai et al., 2013). Among the distinctive characteristics that distinguish each phase, the accumulation of secondary metabolites is of great interest with more than 300 individual phenolic and flavonoid compounds reported being abundant in grapes and wine (Šikuten et al., 2020; Darwish et al., 2021).

The economic value of grapes draws significant attention to manifest all grape-related aspects, emphasizing berry development. Since the genome of grapevine (*Vitis vinifera*) has been sequenced and annotated (Jaillon et al., 2007), several studies have been conducted to better elucidate the transcriptomic and metabolic reprogramming that govern berry development (Deluc et al., 2007; Nwafor et al., 2014; Palumbo et al., 2014; Degu et al., 2015). In contrast, muscadine received less recognition with relatively few high-throughput studies (Pinu, 2018; Duan et al., 2019). The recent release of muscadine whole genome sequence has facilitated transcriptomic studies associated with several berry quality aspects (Park et al., 2021). In our previous study, we utilized an untargeted metabolomics approach along with multivariate analysis to categorize the metabolome profile throughout the six berry developmental stages of three muscadine grape genotypes: the bronze cultivar Late Fry (LF) and two-colored breeding lines, C5-9-1 (C5) and C6-10-1 (C6) (Darwish et al., 2021). The data illustrated that the differences in total phenolic content (TPC) and total flavonoid content (TFC) levels resulted in alterations in DPPH, FRAP, and ABTS antioxidant activities. However, the véraison (V), postvéraison (PoV), and ripening developmental stages were the most distinguishing stages between genotypes. Given the changes in TPC and TFC levels during development, the following question was raised: what is the crucial gene network that can cause a difference in phenolic/flavonoid accumulation, and hence the antioxidant activity of muscadine grape. To answer this question, two contrasting muscadine genotypes (C5 and C6) with varied phenolic/flavonoid content and antioxidant activity were investigated. The changes in TPC/TFC levels and antioxidant activity during berry development along with RNA-sequencing (RNA-seq) strategy were utilized to identify the molecular events associated with phenolic/flavonoid accumulation. The results showed that berry development is concomitant with modulation of transcriptomic profile changes that were more pronounced at the V stage. Despite that, the downregulation model of gene expression prevailed over the upregulation pattern during the progression in berry development, the expression levels were highly pronounced in C5 during prevéraison (PrV), V, and PoV stages. Of paramount significance, the genes encoded key enzymes involved in the modification reactions of polyphenolics biosynthetic pathway, including hydroxylation, methylation, and glycosylation were more abundant during the V stage of C5 genotype than C6, defining the high accumulation of bioactive phenolic/flavonoid derivatives in C5.

## Materials and Methods

### Plant Materials

**FIGURE 1:**
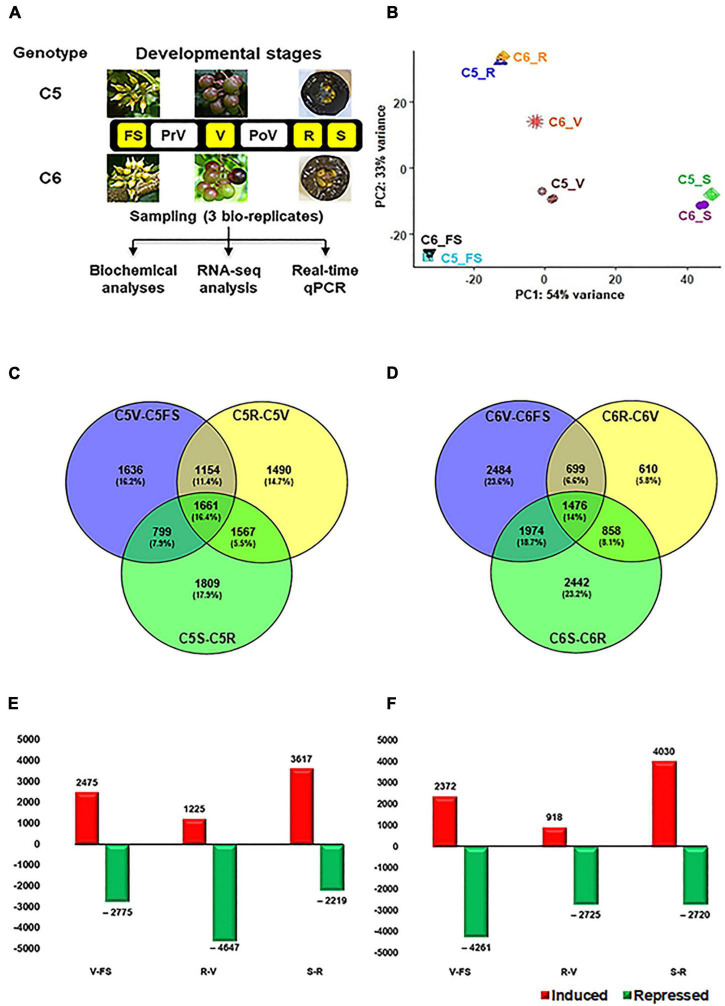
Overall experimental procedure of muscadine berries. **(A)** The layout of the study used the two muscadine genotypes, C5 (C5-9-1) and C6 (C6-10-1), exhibiting differential phenolic/flavonoid levels and antioxidant activity profiles (Darwish et al., 2021). The developmental stages highlighted in yellow represent the samples selected for RNA-seq, including fruit-set (FS), véraison (V), ripe skin/flesh (R), and ripe seeds (S). However, pre- and postvéraison stages (PrV and PoV) were only included in the quantitative PCR (qPCR) assay. **(B)** The principal components analysis (PCA) of the samples’ replicates, where normalized counts were transformed to the variance stabilizing transformation (VST) using DESeq2. Each unique combination of a developmental stage was given a distinctive color. **(C,D)** By using DESeq2 and EdgeR pipeline, each stage was compared to its preceding one within the same genotype. As a result, 10,116 and 10,543 non-redundant differentially expressed genes (DEGs) were identified in C5 and C6, respectively, with fold2change > ± 1.5. **(E,F)** Bar plot of DEGs in C5 and C6 when each stage was compared to its former one.

The berry samples were collected from 5-year-old muscadine grapevine (*Muscadine rotundifolia* (Michx.) small) genotypes C5-9-1 (C5) and C6-10-1 (C6), grown at the experimental vineyard of the Florida A&M University (Tallahassee, FL, United States). These two breeding lines were developed under the grape breeding program of the Center for Viticulture and Small Fruit Research. They were selected according to their diversity in TPC, TFC, and antioxidant activity (Mendonca et al., 2019; Darwish et al., 2021). The berry samples were collected at different developmental stages, including fruit-set (FS), PrV, V, PoV, and ripening (Figure 1A and Supplementary Table 1). The berries were carefully separated into two different tissues at the ripening stage, designated as ripe skin/flesh (R) and ripe seeds (S) tissues. Five clusters/replicate and three replicates/genotype were randomly collected for all developmental stages, excluding the FS stage. At FS, 70 clusters/replicates were collected due to the small berry size. All samples were immediately frozen in liquid nitrogen and stored at –80°C for further analysis.

### Nucleic Acid Extraction and RNA-seq Library Construction

The total RNA from the muscadine berry tissues was extracted as described previously (Gambino et al., 2008). All RNA extracts were treated with the RNase-Free DNase Set (Qiagen, Valencia, CA, United States), and then cleaned up with the RNeasy Mini Kit (Qiagen, Valencia, CA, United States). A total of 24 RNA-seq libraries (three biological replicates at four stages, FS, V, R, and S) from the C5 and C6 muscadine genotypes were constructed as described previously (Bai et al., 2014) using the NEBNext Ultra II RNA Library Prep Kit for Illumina (New England Biolabs, Ipswich, MA, United States). These libraries were multiplexed in an equal amount for paired-end 150-base sequencing in two lanes of NovaSeq 6000 (Illumina, San Diego, CA, United States) at the Novogene Co., Ltd. (Sacramento, CA, United States).

### RNA-seq Data Preprocessing and Identification of Differentially Expressed Genes

The Illumina sequencing of the multiplexed RNA-seq libraries yielded 24 FASTQ files of sequences. Reads quality was checked by FASTQ^1^ two times before and after trimming using Trimmomatic v0.39 (Supplementary Table 2; Bolger et al., 2014). The trimmed reads were then subjected to Salmon in non-alignment-based mode to estimate transcript quantification (Patro et al., 2017). All the samples mapped to the novel generated transcriptome of muscadine grape yielded a mapping rate higher than 77.1% (Supplementary Table 2; Park et al., 2021). Differentially expressed genes (DEGs) during berry development were identified between consecutive developmental stages [V-FS, R-V, or seeds – R (S-R)] within the C5 or C6 genotype, and between analogous stages of C5 and C6, using DESeq2 and EdgeR package setting on default parameters (Love et al., 2014; Chen et al., 2016). For convenience, the common and unique DEGs of each comparison generated by DESeq2 and EdgeR pipeline (DEGs, *P*_FDR_ < 0.05, log2fold change > 1.5 or < –1.5) were considered to be expressed (Supplementary Figures 3–5 and Supplementary Tables 3–5). The web-based tool Venny was used to construct the consensus result (Oliveros, 2007). Finally, *K*-means clustering was performed with the Hartigan-Wong Algorithm and based on inputs of log2 relative Transcripts Per Million (TPM) values.

### Weighted Gene Co-expression Network Analysis

The co-expression network modules were constructed using the variance stabilizing transformation values and the R package WGCNA (v1.69; Langfelder and Horvath, 2008). Before analyzing the data, lowly expressed genes among all sample types were removed by DESeq2, and the remaining 20,886 genes were used in module construction. The co-expression modules were obtained using the default settings, except that the soft threshold power was 10, TOMType was unsigned, minModuleSize was 30, mergeCutHeight was 0.25, and scale-free topology fit index was 0.8 (*R*^2^ = 0.8). A module eigengene (ME) value, which summarizes the expression profile of a given module as the first principal component, was calculated and used to evaluate the association of modules with berries biochemical property [TPC, TFC, and total anthocyanin content (TAC)] and antioxidant activity [DPPH, FRAP, ABTS, NORS, and CUPRAC] of C5 and C6 genotypes at different developmental stages (Darwish et al., 2021). As a result, the final matrix for WGCNA contained a total of 20,886 genes that were assigned to sixteen modules (M1-M16). In addition, the number of non-redundant DEGs from C5_stages_, C6_stages_, and C5_stage_ - C6_stage_ comparisons, which are present in each module, were determined.

### Gene Ontology Enrichment and Kyoto Encyclopedia of Genes and Genomes Pathway Analyses

The gene ontology (GO) and Kyoto Encyclopedia of Genes and Genomes (KEGG) enrichment analyses were assigned using the g:Profiler website by applying the Benjamini–Hochberg multiple testing correction method with *P*_FDR_ < 0.05 (Raudvere et al., 2019). However, since the gene ID of muscadine transcriptome is not supported, the Ensembl gene ID of *Vitis vinifera* was used instead. The Cytoscape plug-in ClueGO was used to visualize the non-redundant BP terms and KEGG pathways for DEGs located in modules ME1, ME5, ME11, and ME14, as well as the 94 genes of interest (Bindea et al., 2009).

### Validation of Differentially Expressed Genes Subsets by Quantitative PCR

The DNase-treated RNA (5 μg) was reverse transcribed in a reaction of 50 μl using the High Capacity cDNA Reverse Transcription Kit (Applied Biosystems, Foster City, CA, United States). The gene-specific primers were designed using Primer Express (v3.0, Applied Biosystems, Foster City, CA, United States) (Supplementary Table 11). The real-time quantitative PCR (qPCR) assays were performed using 20 ng of cDNA and 300 nM of each primer in a 10-μl reaction volume with SsoAdvanced Universal SYBR Green Supermix (Bio-Rad Laboratories, Hercules, CA, United States). Three biological and three technical replicates for each reaction were analyzed on a CFX384 Touch Real-Time PCR Detection System instrument (Bio-Rad Laboratories, Hercules, CA, United States) with the first step of 95°C for 5 min followed by 40 cycles of 95°C for 10 s, 60°C for 10 s, and 72°C for 20 s. Melting curves were generated using the following program: 95°C for 15 s, 60°C for 15 s, and 95°C for 15 s. Transcript abundance was quantified using standard curves for the target and reference genes, generated from serial dilutions of PCR products from corresponding cDNAs. The transcript abundance was normalized to the reference genes *MrActin* and *MrEF1*, which showed high stability across the different muscadine genotypes and tissues used. The geometric mean of the selected housekeeping genes was validated as an accurate normalization factor.

## Results

### Global Changes in Muscadine Transcriptome Through Berry Development

Our previous work built an extensive classification of metabolome profile throughout the six berry developmental stages of contrasting C5 and C6 muscadine genotypes, showing an apparent reduction in the TPC and TFC along with the progression in berry development. However, the differences between the two genotypes were manifested during the developmental stages of V, PoV, and ripening (R). The C5 genotype displayed higher TPC and TFC levels, hence more pronounced DPPH and FRAP antioxidant activities were detected (Darwish et al., 2021). To identify the molecular events associated with metabolome accumulation during berry development, the RNA-Seq strategy was used to generate transcriptome profiles with target berry developmental stages (FS, V, R, and S) from C5 and C6 genotypes cultivated under the same conditions (Figure 1A). Even though they are complex reproductive organs, the ripe berries’ seeds were considered as a fruit tissue/stage. After removing the low-quality reads, 18.8–30.5 million high-quality clean reads per replicate were acquired, which have been deposited in NCBI GenBank (PRJNA775666). The reads from the 24 libraries were mapped against the muscadine transcriptome with a 77.1–80.5% mapping rate (Supplementary Table 1). The principal component analysis (PCA) showed high consistency among transcript profiles and a clear separation of two main components. The first component (PC1) was accountable for 54% of the variance and associated with the berry development procedure. However, the second component (PC2) was responsible for 33% of the variance and mainly associated with genotype disparity, most notably at the V stage (Figure 1B). Progressive changes to the transcript abundance were more evident when the data were subjected to hierarchical clustering (Supplementary Figure 1).

To identify the DEGs during berry development, the data were subjected to two different statistical packages, DESeq2 and EdgeR (Love et al., 2014; Chen et al., 2016). The DEGs (*P*_FDR_ < 0.05, log2fold change > 1.5 or < –1.5) of each comparison generated by DESeq2 or EdgeR pipelines were considered (Supplementary Figures 2, 3 and Supplementary Tables 2, 3). Three pairwise transcriptome comparisons between consecutive berry developmental stages (V-FS, R-V, or S-R) within C5 (C5_stages_) and C6 (C6_stages_) genotypes resulted in 10,116 and 10,543 non-redundant DEGs, respectively (Figures 1C–F). In general, a number of 8518 genes were commonly upregulated or downregulated within the stages of both genotypes (Supplementary Figure 4). The data manifested that berry development involves transcriptional reprogramming of a large number of genes. In C5, there was a slight variation in the total and exclusive number of DEGs signified in each comparison (Figure 1C). However, the number of upregulated and downregulated transcripts considerably varied with the progression in berry development. This was most apparent at the V stage, exhibiting a ∼4-fold higher number of upregulated transcripts than in the R stage (C5_R–V_, Figure 1E and Supplementary Table 2). In C6, the total and exclusive numbers of DEGs in the C6_R–V_ comparison were ∼2- and ∼4-fold lower relative to other comparisons, respectively (Figure 1D). However, the number of upregulated transcripts during the V stage was ∼3-fold higher than the R stage (C6_R–V_), but roughly 50% of the transcripts during the FS stage (C6_V–FS_, Figure 1F and Supplementary Table 3). The data suggested that the downregulation pattern prevailed the upregulation profile of genes expression throughout berry development, particularly within C6 stages, confirming the previously reported results in different *Vitis vinifera* cultivars (Fasoli et al., 2012; Massonnet et al., 2017). In addition, the V stage harbored the most pronounced transcriptional modulation, especially within C5 stages.

### Véraison Signifies the Main Divergent Between Genotypes During Development

**FIGURE 2:**
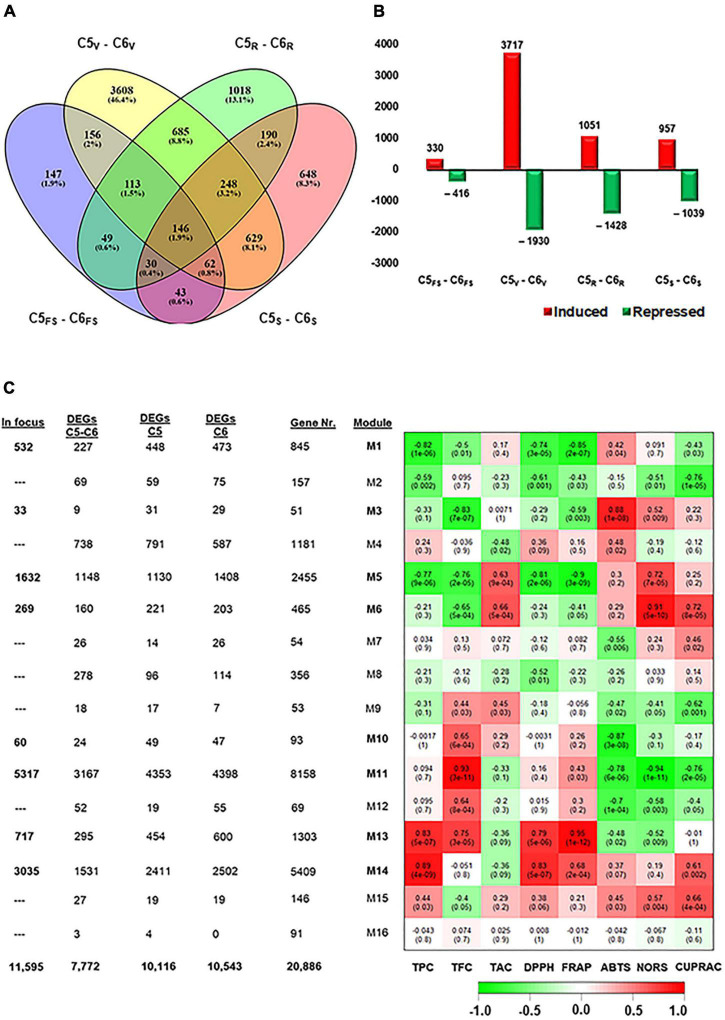
Differentially expressed genes among C5 stages and their corresponding stages in C6. **(A,B)** Venn diagrams and bar plots of DEGs resulted from comparing equivalent stages in C5 and C6 genotypes, using DESeq2 or EdgeR pipelines. As a result, 7772 non-redundant DEGs were identified with log2fold change > 1.5 or < –1.5. **(C)** Module-trait associations between RNA-seq data and evaluated biochemical-related traits, including TPC, TFC, total anthocyanin content (TAC), and different types of antioxidant assays (DPPH, FRAP, ABTS, NORS, and CUPRAC) from the C5 and C6 genotypes at different berry developmental stages. The color of the cell indicates the correlation coefficient between a given module and biochemical-related traits at the row-column intersection. Each row corresponds to a module (M1–M16). The left panel shows the assigned number of genes to each module, whether the number of total input genes or DEGs from C5_stages_, C6_stages_, and C5_stage_ – C6_stages_ comparisons. In addition, modules of interest were shown in bold line and selected for further analysis (indicated as in focus). Green and red are the color key that represents *r*^2^ values from –1 to +1. FS, fruit-set; V, véraison; R, ripe skin/flesh; S, ripe seed.

To extend our knowledge of how the transcript abundance pattern differentiates in C5 and C6 during berry development, the data were subjected to the same pipelines described above. The different berry developmental stages in C5 were compared against their analogous in C6 (C5_stage_–C6_stage_). The four pairwise transcriptome comparisons (C5_FS_-C6_FS_, C5_V_-C6_V_, C5_R_-C6_R_, and C5_S_-C6_S_) identified 746, 5647, 2479, and 1996 DEGs, respectively; and 7772 non-redundant transcripts in all comparisons (Figures 2A,B, Supplementary Figure 5, and Supplementary Table 4). In agreement with PCA (Figure 1B), the number of DEGs in the V stage comparison (C5_V_-C6_V_) represented approximately 50% of the total redundant/non-redundant DEGs (Figures 2A,B). Moreover, the number of upregulated transcripts during the V stage in C5 was ∼2-fold higher than in C6, while other C5_stage_-C6_stage_ comparisons exhibited a slight variation. Surprisingly, the data showed that 41% of DEGs (5,372 out of 13,000) in C5_stage_ and C6_stage_ comparisons were shared within the C5_stages_/C6_stages_ comparisons (Supplementary Figure 6). Only 6.6% of DEGs were exclusively present in C5_stage_-C6_stage_ or within C5_stages_ analysis comparing to 9.4% of DEGs within C6_stages_. The C5_stage_-C6_stage_ data agreed with C5_stages_ and C6_stages_ comparison results, confirming that the dramatic transcriptomic changes that differentiate C5 from C6 occurred during the V stage.

### Construction of Gene Co-expression Networks

The WGCNA system biology approach was utilized to construct co-expression networks based on pairwise correlations of non-lowly expressed genes of the 24 samples. Sixteen modules were identified and labeled in distinct colors shown in a hierarchical clustering dendrogram and network heatmap (Supplementary Figures 7, 8). Next, we performed the analysis of module-trait correlations between the 16 modules and the biochemical property data (TPC, TFC, and TAC) or antioxidant activity data (DPPH, FRAP, ABTS, NORS, and CUPRAC) from both genotypes over berry developmental stages (ME1–ME16, Figure 2C). Out of the 16 modules, eight modules showed significant correlations with the trait data. To link the WGCNA modules with the previously analyzed data (C5_stages_, C6_stages_, and C5_stage_-C6_stage_), the acquired DEGs from each analysis were assigned to each module (Figure 2C and Supplementary Figure 9). Hence, our focus was restricted to the 11,595 non-redundant DEGs located in modules potentially associated with the phenolic/flavonoid accumulation and the antioxidant activity, including ME1, ME3, ME5, ME6, ME10, ME11, ME13, and ME14 (Supplementary Table 5). The ME1, ME5, and ME10 were negatively correlated modules holding 532, 1632, and 60 DEGs, respectively. Notably, ME1 and ME5 displayed a substantial negative correlation with TPC, DPPH, and FRAP (*r*^2^ ≥ –0.74). However, the ME13 and ME14 modules involving 717 and 3035 DEGs were positively correlated with TPC and DPPH (*r*^2^ ≥ 0.79). The ME13 further showed a positive correlation with TFC and FRAP (*r*^2^ ≥ 0.75). The ME3, ME6, and ME11 modules exhibited a distinct correlation profile of both negative and positive correlations. For instance, the ME11 was the most extensive module with 5317 DEGs that were positively correlated with TFC (*r*^2^ = 0.93) but negatively correlated with the ABTS, NORS, and CUPRAC (*r*^2^ ≥ –0.76). A detailed description of the module-trait correlations is presented in Figure 2C.

To provide a broad overview of the types of DEGs located in the modules of interest, GO terms and KEGG enrichment analyses were performed based on *V. vinifera* Ensembl GeneID (Raudvere et al., 2019; Supplementary Table 6). Among the eight modules of interest, four modules (ME1, ME5, ME11, and ME14) exhibited high enrichment in GO terms in the molecular function (MF) for transferase and oxidoreductase activities. Moreover, ME1 and ME11 were highly enriched in GO terms belonging to the biological process (BP) for flavonoid metabolic process (GO:0009812) and phenylpropanoid metabolic process (GO:0009698), respectively (Supplementary Figures 10, 12). Similarly, ME1, ME5, ME11, and ME14 were highly enriched in the KEGG pathway for isoflavonoid (vvi00943), diterpenoid (vvi00904), phenylpropanoid (vvi00940), and terpenoid (vvi00900) backbone biosynthesis, respectively (Supplementary Figures 10–13 and Supplementary Table 6). Moreover, ME1 and ME5 exhibited significant enrichment for flavonoid biosynthesis (vvi00941). To identify the major transcriptional dynamics associated with berry development, K-means clustering was performed to partition DEGs within modules of interest into specific behaviors. The 11,595 DEGs were classified into 15 K-means clusters (K1–K15) that were comparable but with different kinetics in both genotypes (Supplementary Figures 14, 15 and Supplementary Tables 7, 8). Interestingly, C5 showed a complete K7 cluster that has no comparable pattern in C6. Temporal expression trends within each module were characterized by building modules/K-clusters Circos. Although the modules were represented by the same number of DEGs, they displayed distinctive profiles between C5 and C6 (Supplementary Figures 16, 17).

### Identification of Genes in the Polyphenolics Pathway

**FIGURE 3:**
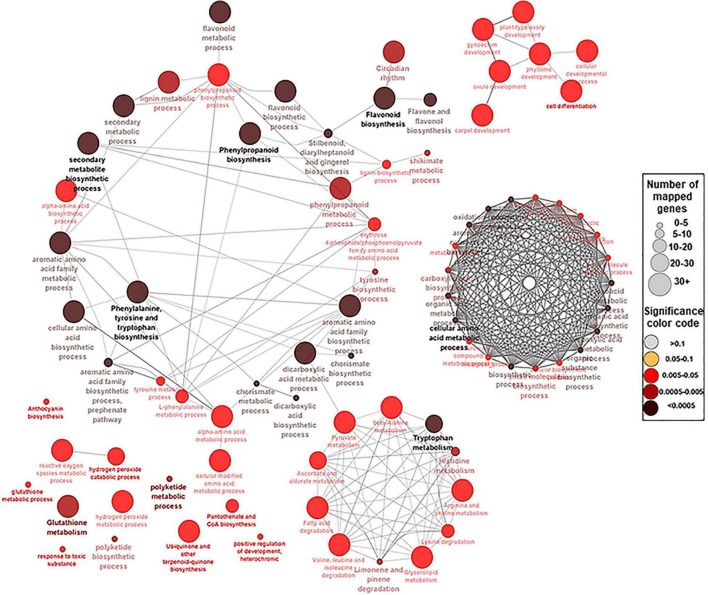
A network view for the predefined biological processes Gene Ontology (GO) terms and Kyoto Encyclopedia of Genes and Genomes (KEEG) pathways for the 94 hub genes. BP GO terms and KEEG pathways for the selected 94 genes (*p*-adjusted < 0.05), extracted by g:Profiler website with Benjamini–Hochberg false discovery rate (FDR) multiple testing correction method. The default ClueGO settings were applied, and the terms are functionally grouped based on shared genes (kappa score). The size of the nodes indicates the number of mapped genes ranged from 0–5, 5–10, 10–20, 20–30, and ≥30 genes. The size of the nodes indicates the number of mapped genes, while the color indicates the degree of significance (0.1 < *p*-value < 0.0005). The most significant term defines the name of the group.

To identify the key genes involved in phenolic/flavonoid biosynthesis, annotation information of DEGs within modules of interest was extracted from the *de novo* muscadine reference genome annotation (Park et al., 2021). A number of 94 genes were identified based on their predicted functions (Supplementary Table 9). These hub genes were located in six modules (ME1, ME5, ME6, ME11, ME13, and ME14) and different K-means clusters (Supplementary Figure 18). The GO term and KEGG analyses among the candidate genes showed significant enrichment of biological pathways for shikimate metabolic process, glutathione metabolism, drug transmembrane transport, and drug transport, along with flavonoid, flavone, flavonol, phenylalanine, phenylpropanoid, anthocyanin, stilbenoid, and lignin biosynthetic process (Figure 3 and Supplementary Table 10).

**FIGURE 4:**
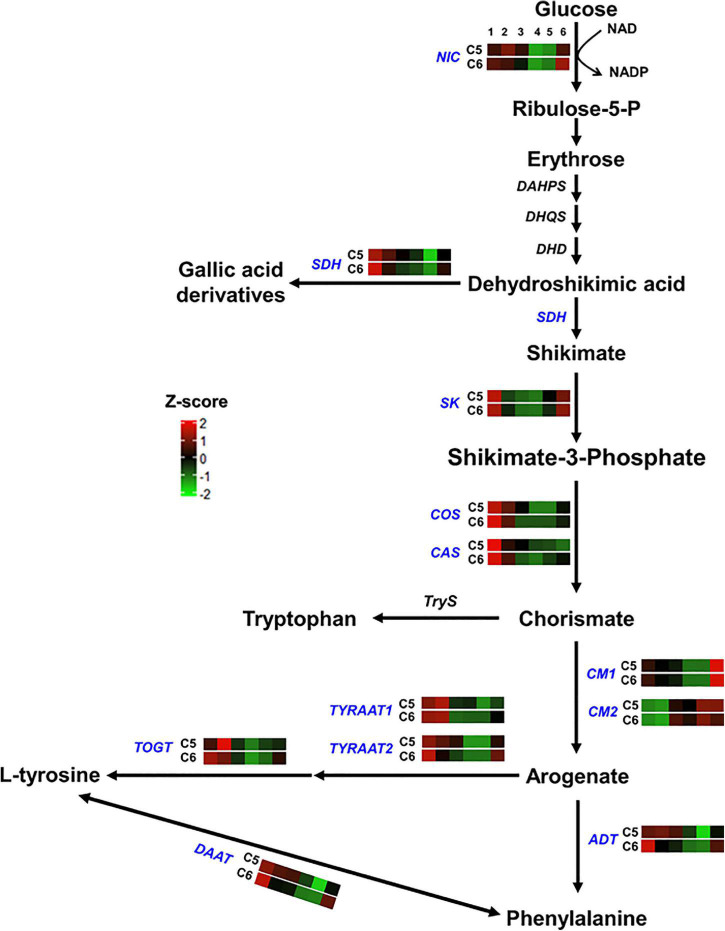
Shikimic and amino acids pathway. The colored boxes show the accumulation pattern of genes involved in shikimic and amino acids pathway that were assessed by qPCR at different berry developmental stages, including fruit-set (1), prevéraison (2), véraison (3), postvéraison (4), ripe skin/flesh (5), and ripe seeds (6). Red boxes indicate higher levels of expression, and green boxes indicate lower expression levels. The color brightness is directly proportional to the expression ratio, according to the color scale. *NIC*, nicotinamidase; *SDH*, shikimate dehydrogenase; *SK*, shikimate kinase; *COS*, chorismate synthase; *CAS*, caffeine synthase; *CM*, chorismate mutase; *TYRAAT*, arogenate dehydrogenase; *TOGT*, tyrosine biosynthetic process; *ADT*, arogenate dehydratase/prephenate dehydratase; *DAAT*, *D*-amino-acid transaminase.

**FIGURE 5:**
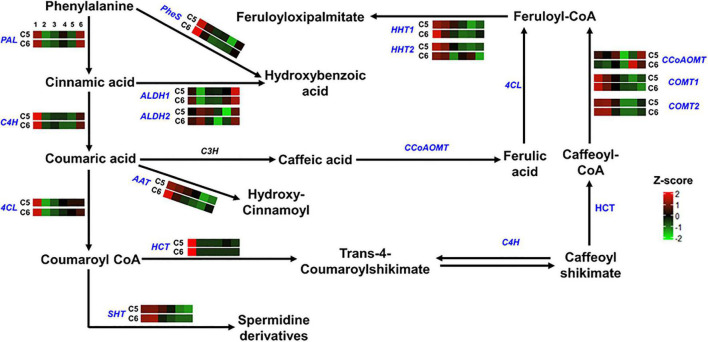
Hydroxycinnamic acids pathway. The colored boxes show the accumulation pattern of genes involved in hydroxycinnamic acids pathway that were assessed by qPCR at different berry developmental stages, including fruit-set (1), prevéraison (2), véraison (3), postvéraison (4), ripe-skin/flesh (5), and ripe-seeds (6). Red boxes indicate higher levels of expression, and green boxes indicate lower expression levels. The color brightness is directly proportional to the expression ratio, according to the color scale. *PAL*, phenylalanine ammonia-lyase; *C4H*, trans-4-coumarate biosynthesis; *4CL*, 4-coumaroyl:CoA-ligase; *PheS*, phenylalanine ligase; *ALDH*, aldehyde dehydrogenase; *AAT*, anthocyanidin 3-*O*-glucoside 6″-*O*-acyltransferase; *HCT*, shikimate *O*-hydroxycinnamoyl transferase; *SHT*, spermidine hydroxycinnamoyl transferase; *HHT*, omega-hydroxypalmitate *O*-feruloyl transferase; *CCoAOMT*, caffeoyl-CoA *O*-methyltransferase; *COMT*, caffeic acid 3-*O*-methyltransferase.

**FIGURE 6:**
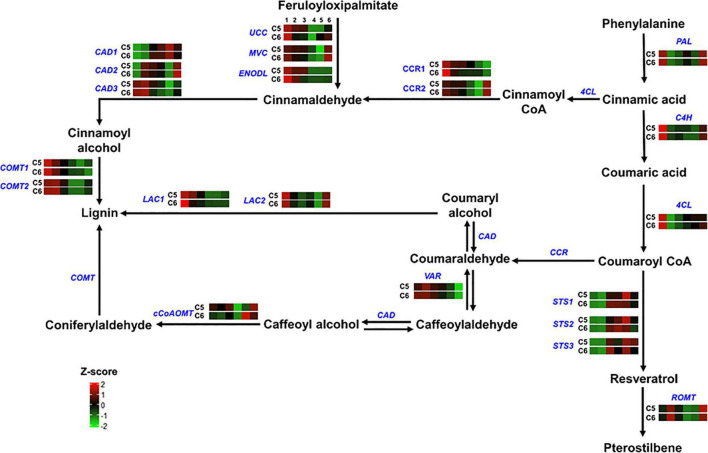
Lignin and pterostilbene pathway. The colored boxes show the accumulation pattern of genes involved in lignin and pterostilbene pathway that were assessed by qPCR at different berry developmental stages, including fruit-set (1), prevéraison (2), véraison (3), postvéraison (4), ripe-skin/flesh (5), and ripe-seeds (6). Red boxes indicate higher levels of expression, and green boxes indicate lower expression levels. The color brightness is directly proportional to the expression ratio, according to the color scale. *UCC*, uclacyanin-3; *MVC*, mavicyanin; *ENODL*, early nodulin; *CAD*, cinnamyl alcohol dehydrogenase; *CCR*, cinnamoyl-CoA reductase; *VR*, vestitone reductase; *LAC*, laccase; *STS*, stilbene synthase; *ROMT*, trans-resveratrol di-O-methyltransferase. Underlined genes, including *PAL*, *C4H*, *4CL*, and *CCoAOMT* are shown in Figure 5.

**FIGURE 7:**
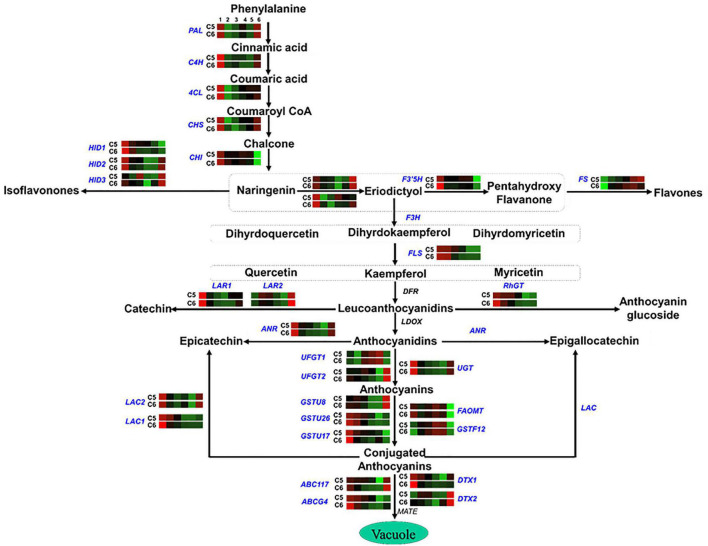
Flavonoid pathway. The colored boxes show the accumulation pattern of genes involved in flavonoid pathway that were assessed by qPCR at different berry developmental stages, including fruit-set (1), prevéraison (2), véraison (3), postvéraison (4), ripe-skin/flesh (5), and ripe-seeds (6). Red boxes indicate higher levels of expression, and green boxes indicate lower expression levels. The color brightness is directly proportional to the expression ratio, according to the color scale. *CHS*, chalcone synthase; *CHI*, chalcone isomerase; *HID*, 2-hydroxyisoflavanone dehydratase; *F3H*, flavonoid 3′-monooxygenase; *F3′5′H*, flavonoid 3′5′-hydroxylase; *FS*, flavonol synthase; *FLS*, flavonol sulfotransferase; *LAR*, leucoanthocyanidin reductase; *RhGT*, anthocyanidin 5,3-*O*-glucosyltransferase; *ANR*, anthocyanidin reductase; *UGT*, gallate 1-beta-glucosyltransferase; *UFGT*, anthocyanidin 3-*O*-glucosyltransferase; *OMT/FOMT*, flavonoid 3,5-methyltransferase; *GST*, glutathione S-transferase; *ABC*, ATP-binding cassette transporter; *DTX*, protein detoxification.

**FIGURE 8:**
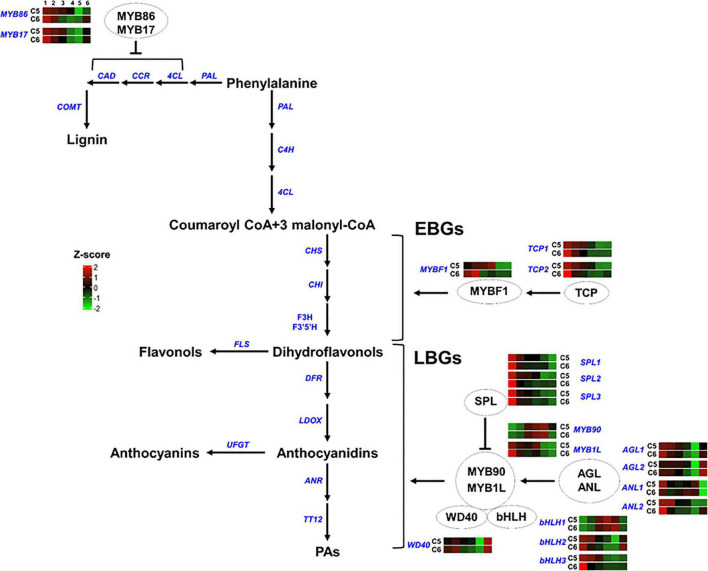
Transcription factors regulating the phenylpropanoid/flavonoid pathway. The colored boxes show the accumulation pattern of genes involved in the regulation of flavonoid and lignin pathway that were assessed by qPCR at different berry developmental stages, including fruit-set (1), prevéraison (2), véraison (3), postvéraison (4), ripe-skin/flesh (5), and ripe-seeds (6). Red boxes indicate higher levels of expression, and green boxes indicate lower expression levels. The color brightness is directly proportional to the expression ratio, according to the color scale. *MYB*, myb-related regulatory gene; *TCP*, teosinte branched1/Cincinnata/proliferating cell factor; *SPL*, squamosa promoter-binding-like protein; *AGL*, agamous MADS-box protein; *ANL*, homeobox-leucine zipper protein anthocyaninless; *bHLH*, basic helix-loop-helix; *WD40*, tryptophan-aspartic acid repeat. EBGs and LBGs for early and late biosynthetic genes, respectively.

To validate the expression patterns and identify the crucial candidate genes underlying the diversity in phenolic/flavonoid accumulation and antioxidant activity in both genotypes, we performed qPCR to the hub genes at different developmental stages. Since the variance associated with the genotype (PC2) was remarkably evident at the V stage (Figures 1A,B), we decided to include the PrV and PoV stages in the experiment. The qPCR results of selected genes at FS, V, R, and S stages were significantly correlated (*r*^2^ ≥ 0.933, *P* < 9.8 × 10^–7^) with their TPM values, confirming the RNA-seq expression data s (Supplementary Table 11). For convenience, the 94 genes were classified into six groups based on their location in the phenolic/flavonoid biosynthesis (Figures 4–8). Generally, all genes showed comparable expression patterns but with different kinetics between genotypes.

For genes involved in the shikimic and amino acids pathway (group-1), 3 out of 12 genes showed distinct accumulation patterns, including the nicotinamidase (*NIC*), the arogenate dehydratase/prephenate dehydratase (*ADT*), and the UDP-phenylpropanoid glucosyltransferases (*TOGT*) (Figure 4 and Suppplementary Figure 19). Relative to C6, *NIC* transcripts steadily increased to a peak at the PrV stage. The expression level of *NIC* was ∼2- and ∼3-fold higher in C5 at the PrV and V stages. Similarly, the abundance of *ADT* was visibly higher in C5 (∼9.5-fold) at the same stages. Interestingly, a strong peak signal of *TOGT* was detected at the PrV stage of C5, exhibiting ∼175-fold higher transcript abundance and sharply decreased afterward. In general, all genes related to the shikimic and amino acids pathway were more abundant in C5 than in the C6 genotype, particularly at the PrV and V stages, excluding the chorismate mutase2 gene (*CM2*).

The comparable accumulation pattern was also noted for gene-encoded enzymes involved in the hydroxycinnamic acids pathway (group-2). Only two out of the 14 genes coordinating the pathway exhibited differential expression profiles between the two genotypes (Figure 5 and Supplementary Figure 20). The omega-hydroxypalmitate *O*-feruloyltransferase2 (*HHT2*) expression displayed a shifted pattern in C6 genotype toward induction during the late stages of PoV and R. However, the expression pattern of the caffeoyl-CoA *O*-methyltransferase (*CCoAOMT*) was indistinguishable between the two genotypes before it peaked by ∼6.5-fold at the R stage of C6.

Regarding the lignin and pterostilbene pathway (group-3), the cinnamyl alcohol dehydrogenase 1 (*CAD1*) and the vestitone reductase (*VR*) genes displayed high accumulation levels that occurred mainly at the PrV stage, reaching ∼7.5- and ∼3.5-fold higher in C5 genotype, respectively (Figure 6 and Supplementary Figure 21). However, this pattern was inversed in the case of stilbene synthase genes (*STS1-3*) that manifested higher levels at the V, PoV, and R stages of C6, suggesting the intense activation of the pterostilbene pathway during the late developmental stages of the C6 genotype.

In the flavonoids/anthocyanins pathway (group-4), several genes exhibited similar accumulation profiles between the two genotypes, considering that both genotypes are colored, albeit higher transcription levels were detected in C5 during PrV, V, and PoV stages such as chalcone isomerase (*CHI*), 2-hydroxy isoflavanone dehydratase (*HID1/2*), flavonoid 3′-monooxygenase 1 (*F3H1*), flavonoid 3′5′-hydroxylase (*F3′5′H*), flavonol sulfotransferase (*FLS*), leucoanthocyanidin reductase 1 (*LAR1*), anthocyanidin 5,3-*O*-glucosyltransferase-like (*RhGT*), anthocyanidin reductase (*ANR*), anthocyanidin 3-*O*-glucosyltransferase (*UFGTs*), glutathione S-transferase (*GSTU17*), and ABC-transporter (*ABCs*) (Figure 7 and Supplementary Figure 23). However, other genes showed a genotype-specific pattern, manifesting remarkable transcription levels at one or more developmental stages of C5 genotype, including chalcone synthase (*CHS*), 2-hydroxy isoflavanone dehydratase 3 (*HID3*), flavonoid 3′-monooxygenase 2 (*F3H2*), leucoanthocyanidin reductase 2 (*LAR2*), and flavonoid 3,5-methyltransferase (*FOMT*). Additionally, the detoxification efflux carrier genes (*DTX1/2*) responsible for the removal of toxins from the cytosol were also higher in C5 (He et al., 2010). However, the flavonol synthase (*FS*) displayed significantly higher levels in C6, excluding the seeds. These data revealed that the expression of genes associated with flavonoids/anthocyanins biosynthetic pathway mainly during the PrV, V, and PoV stages is probably responsible for the differences between C5 and C6 genotypes, regarding the TPC/TFC accumulation and antioxidant capacity (Darwish et al., 2021).

In group-5, different TF-related genes either acting as negative (*MYB86*, *MYB17*, and *SPLs*) or positive (*TCPs*, *AGLs*, *MYB90*, *ANL2*, *bHLH2/3*, and *WD40*) regulators of the phenylpropanoid/flavonoid pathway generally displayed a similar accumulation pattern between the two genotypes. However, higher mRNA levels were detected in C5 during PrV, V, and PoV stages albeit a few exceptions. They showed a rapid decline in their transcription levels along with berry development. Nevertheless, three TFs displayed strong mRNA signals detected at one or more developmental stages of the C5 or C6 genotype (Figure 8 and Supplementary Figure 23). The major differences between C5 and C6 genotypes were evident during the V, PoV, and R stages, particularly for the MYB-transcriptional activators (*MYBF1* and *MYB1L*), the homeobox-leucine zipper protein anthocyaninless (*ANL1*), and *bHLH1*. The *MYBF1* sharply peaked at the PoV, reaching ∼75-fold higher in C5 compared with C6 that retained at basal low levels after the PrV (Figure 8; Li and Zachgo, 2013). The expression of *MYB1L* was initially high at the FS stage and rapidly declined in both genotypes; however, it exhibited a strong induction, reaching a peak at the PoV stage with ∼9-fold higher in C5. Similarly, the *bHLH1* displayed more abundance at the V stage that continues to a peak at the PoV stage of C5. In C5, *bHLH1* exhibited ∼6.7- and ∼4.8-fold higher than in C6 at corresponding stages, respectively. Interestingly, *ANL1* showed more transcript abundance at the V, PoV, and R stages of C6. Overall, these results highlight genotype-specific trends in the accumulation of the TFs, coordinating the expression of downstream genes associated with the phenolic/flavonoid pathway by which they are abundant mainly during immature developmental stages of both genotypes.

Finally, the genes in group-6 exhibited a similar expression pattern. The peroxidase proteins (*PODs*) are involved in the turnover of vacuolar phenolic metabolites (Barceló et al., 2003). The methanol *O*-anthraniloyltransferase (*AMAT*), the flavin-containing monooxygenase (*FMO*), and the UDP-rhamnose: rhamnosyltransferase (*GRT4s*) are enzymes involved in the production of *O*-methyl anthranilate, formation of the epicatechin 3′-*O*-glucoside, and metabolism of phenolic compounds, respectively (Wang and De Luca, 2005; Nakamura et al., 2012; Hsu et al., 2017). The *PODs*, *AMAT*, *GRT4s*, and *FMO* mRNAs declined rapidly in both genotypes, although pronounced levels were retained in C5 at one or more developmental stages (Supplementary Figure 24). By contrast, one version of the genes encoded the heavy metal transport/detoxification protein (*HMA2*) showed expression induction at the V stage of C5, while the *HMA1* was more abundant in the seeds of both genotypes.

## Discussion

Despite the nutraceutical qualities of muscadine berries, no previous investigation has been conducted to elucidate the molecular basis of potent berry bioactivity. The current investigation provides the first extensive record for mRNA expression profiling in parallel with the changes in TPC/TFC accumulation and antioxidant activity throughout muscadine berry development. The two contrasting muscadine genotypes, C5/C6, were selected based on their diversity in TPC/TFC levels that influence antioxidant capacity (Darwish et al., 2021). The differences between these genotypes exhibiting similar berry color and grown under the same cultivation circumstances were sufficient to identify the critical DEGs coordinating antioxidant bioactivity throughout berry development in a genotype-dependent manner. The data indicated that transcriptional modulation of a large number of genes occurs during berry development. The PCA of the entire transcript profiles showed a clear separation among berry developmental stages. Likewise, the berry transcriptome of different white and red berry grape varieties exhibited a similar extensive shift along with development (Massonnet et al., 2017). Most importantly, the PC2 pointed out the V stage as a decisive event when the separation between genotypes occurred in terms of the levels of TPC/TFC and antioxidant activity. Indeed, the number of upregulated transcripts during C5_V_ was higher than in C6_V_. Furthermore, comparisons among stages showed that the number of upregulated transcripts during the V stage was higher than through the R stage. The data demonstrated the predominance of downregulation mode of expression during berry development, but not for the seed (S) tissue, which is consistent with previous results using different grape cultivars (Fasoli et al., 2012; Massonnet et al., 2017). This downregulation profile was more pronounced within C6_stages_. Moreover, the most transcriptional modulation between C5 and C6 arises during the V stage, affecting mainly phenolic/flavonoid accumulation. Therefore, the piling up of phenolic/flavonoid and antioxidant activity appeared to be directly associated with the transcriptomic modulation of berry development, initiated at the V stage of C5. By contrast, the seeds did not display such considerable differences between genotypes.

In general, berry development is accompanied by alterations in secondary metabolites accumulation, considering it as one of the main factors influencing berry quality. However, the relationship between the phenolic/flavonoid content and muscadine grapes has not been explored yet at a molecular level. The transcriptome profile of the muscadine genotypes during berry development emphasized the remarkable transcriptional shift at the V stage. The identified DEGs output of C5_stages_, C6_stages_, and C5_stage_-C6_stage_ comparisons are highly informative about phenolic/flavonoid related genes that become strongly expressed and co-regulated during berry development. Therefore, in parallel with K-means clustering, we engaged the WGCNA approach to provide significant insight into the spatiotemporal dynamics underpinning differences in gene expression associated with contrasting phenolic/flavonoid content and antioxidant activity (Langfelder and Horvath, 2008). We identified eight out of 16 modules that were highly correlated with the examined traits. Interestingly, not all modules exhibited a positive association. The negatively correlated modules (i.e., ME1, ME5, and ME10) were of great interest since the reduction tendency of gene expression and phenolics accumulation prevailed during berry development (Fasoli et al., 2012; Massonnet et al., 2017; Darwish et al., 2021). To demonstrate the phenotype-genotype variation between C5 and C6, our focus was restricted to the 11,595 non-redundant DEGs located in these modules of interest. The GO and KEGG analyses confirmed the successful application of the WGCNA in analyzing the multivariate data as reported in different biological contexts (Li et al., 2019; García-Gómez et al., 2020; Wang et al., 2020). Only four modules (ME1, ME5, ME11, and ME14) were highly enriched in GO term in the BP and KEGG pathways for phenylpropanoid, flavonoid, isoflavonoid, and terpenoid backbone biosynthesis. Furthermore, we identified 94 phenolic/flavonoid biosynthetic and signaling genes located in ME1, ME5, ME6, ME11, ME13, and ME14, showing different K-means clusters. As expected, these hub genes were strongly enriched in flavone, flavonol, phenylalanine, anthocyanin, stilbenoid, lignin biosynthesis, and shikimate metabolic process, in addition to the previously stated overrepresented GO and KEGG pathways within the modules of interest. The GO and KEGG analyses supported the narrow-down strategy, offering biological knowledge about the hub genes (Consortium, 2004). Some of these overrepresented GO and KEGG pathways were reported during fruit development (Berdeja et al., 2015; García-Gómez et al., 2020). Despite the decline profile of gene expression, the C5 genotype held higher transcription levels of most of these genes mainly during the V stage, justifying the higher TPC/TFC content and antioxidant capacity.

The transcriptome survey identified both common and unique molecular events that characterize the action of muscadine berry development. One of the most distinctive molecular incidents between C5 and C6 was the massive activation of phenolic/flavonoid-related transcripts. Generally, both genotypes exhibited comparable expression patterns but with different kinetics, excluding seed tissue. Of paramount importance, the genes with a contrasting accumulation profile between genotypes expose the contribution of the genotypic diversity factor. For instance, the shikimic and amino acids pathway in C5 was emphasized by the expression of *NIC*, *ADT*, and *TOGT* mRNAs. The NIC encodes a nicotinamidase enzyme that converts nicotinamide to nicotinic acid, representing the most similar homolog of the 2,3-dihydro-2,3-dihydroxybenzoate synthase (EntB) (Hunt et al., 2007). The EntB is involved in a sequential step, leading to the conversion of the isochorismate to the 2,3-dihydroxybenzoic acid (2,3-DHBA) and shikimic acid in plants (Widhalm and Dudareva, 2015). TOGT encodes a phenylpropanoid glucosyltransferase functions mainly on hydroxycoumarin glycosylation (Fraissinet-Tachet et al., 1998). The *TOGT*-suppressed plants displayed a significant reduction in scopoletin glycosylation, the essential process for accumulating phenylpropanoids (Chong et al., 2002). Furthermore, the ADT and TYRAATs catalyze the final steps in phenylalanine and tyrosine biosynthesis, respectively (Rippert et al., 2009). The conversion of phenylalanine to cinnamic acid is the admission point of the phenylpropanoid pathway, where the latter serves as the substrate for most hydroxycinnamic acid derivatives (Alam et al., 2016). Certainly, the expression level of general phenylpropanoid-related biosynthetic genes, including *PAL* and *4CL* was upregulated in C5. Similarly, the *CAD1* and *VR* that are required for monolignol and isoflavonoids biosynthesis were more abundant in C5. By contrast, the three *STS* genes were steadily abundant in C6 (Dixon, 1999; Boudet et al., 2003). The higher transcription level of *NIC*, *ADT*, *TOGT*, *CAD1*, and *VR* during the PrV stage, followed by the abundance of *PAL* and *4CL* during V and PoV stages in C5 may reflect their triggering rules that facilitate the stimulated accumulation of the phenylpropanoid derivatives.

Consistently, the C5 genotype retained higher amounts of the TPC/TFC and antioxidant capacity than in C6, albeit the decline behavior along with development (Darwish et al., 2021). The expression profiles of flavonoid/anthocyanin biosynthetic genes simulated these results. Of special interest, those genes showed a genotype-specific pattern, such as *CHS*, *HID3*, *F3H2*, *F3′5′H*, *LAR2*, and *FOMT*. The CHS catalyzes the first step in the flavonoid biosynthesis, while HID enzyme activity is a critical limiting factor in the isoflavonoids biosynthesis (Winkel-Shirley, 2001; Akashi et al., 2005; Du et al., 2010). The F3′H and F3′5′H enzymes are necessary for the biosynthesis of flavonols (quercetin and myricetin), flavan-3-ols (procyanidin and prodelphinidin), and anthocyanins-dependent compounds (cyanidin and delphinidin) (Jeong et al., 2006; Wang et al., 2014). Proanthocyanidins (PAs; also referred to procyanidins or condensed tannins) are made of oligomers and polymers of (epi)catechin units, exhibiting strong antioxidant activity (Prasain and Barnes, 2014). The LAR and ANR are major enzymes that catalyze the formation of PAs (Bogs et al., 2005; Gagné et al., 2009). Moreover, C5 exhibited upregulation of the OMT gene (*FMOT*) involved in the flavonoids methylation. The alteration in the enzymes underlying the modification reactions of the flavonoid pathway, such as methylation and glycosylation reactions can modify and enhance the bioactivity of the flavonoids and their derivatives (Kim et al., 2010). For instance, the O-methylation of hydroxyl groups in flavonoids reduces their reactivity and increases their antimicrobial capacity (Ibrahim et al., 1998). Finally, the flavonoids glycosylation process *via* UFGT results in color-intensive pigments (Boss et al., 1996). In both genotypes, the abundance of *UFGT* was increased toward the R stage, although higher levels were depicted in C5.

The flavonoid biosynthetic genes can be divided into early biosynthetic genes (EBGs), which catalyze the production of dihydroflavonols, and late biosynthetic genes (LBGs) that lead to the biosynthesis of proanthocyanidins and anthocyanins (Ferreyra et al., 2012; Xu et al., 2015). Flavonoid/anthocyanin biosynthesis is coordinated by a transcription complex composed of two transcription factors that belong to the R2R3-MYB and the bHLH-MYC families, and a WD40 cofactor. The three proteins cofunction by establishing the MYB-bHLH-WD40 (MBW) complex to activate the expression of a downstream cascade of flavonoid/anthocyanin structural genes (Yan et al., 2021). Despite the critical contribution of bHLH and WD40 in the complex, the MYB protein is the key component in providing specificity and determining the rate of the subsets of biosynthetic genes (Zimmermann et al., 2004). Evaluation of several mutants provided strong evidence regarding the autonomous role of MYB in mediating the transcription of EBGs that lead to the production of the colorless dihydroflavonols. However, the activation of LBGs that leads to the production of anthocyanins (color pigmentation), requires the MBW complex (Baudry et al., 2004; Stracke et al., 2007; Gonzalez et al., 2008). The MBW complex is regulated by several factors (Li, 2014). The MYB genes that act as negative regulators of the pathway suppress the formation of the MBW complex by competing with R2R3-MYBs for interactions with the bHLH component of the MBW complex (Dubos et al., 2008; Matsui et al., 2008). The squamosa promoter-binding-like protein (SPL) disrupts the MBW complex by competitively binding to the R2R3-MYB subunit of the MBW complex (Gou et al., 2011). By contrast, the positive fine-tuners, including TCP3, organize the formation of the MBW complex by synergistically associating with R2R3-MYBs proteins (Li and Zachgo, 2013). Furthermore, the lignin inhibitor agamous-like protein (AGL) stimulates the flavonoid pathway by maintaining the balance of flavonoid/lignin pathways, redirecting the metabolic flux between the two pathways (Giménez et al., 2015). Finally, the anthocyaninless, a homeodomain protein, controls the accumulation of anthocyanins in subepidermal tissue (Kubo et al., 1999).

The transcription activators *MYBF1*, *MYB90*, and *MYB1L* were strongly accumulated in C5. The MYBF1 acts downstream TCP to stimulate the EBGs pathway (Li and Zachgo, 2013). Consistently, expression of the EBGs, including *CHS*, *CHI*, *F3H*, *F3′5′H*, and *FLS* were more abundant in C5. The MYBF1 is a key flavonol regulator activating the expression of flavonol synthase, and hence accumulates flavonol at early stages of development (Czemmel et al., 2009). However, the earlier and higher levels of *FS* in C6 suggested undetected version(s) of *FS* in muscadine transcriptome. In *V. vinifera*, the *VvMYBPA1*, *VvMYBPA2*, and *VvMYB5a* are required for triggering the EBGs (Czemmel et al., 2012). The *VvMYBPA1* is considered a key regulator of PAs synthesis, controlling the general flavonoid pathway and the PAs branch genes ANR and LAR (Bogs et al., 2007; Gagné et al., 2009). Interestingly, none of these TFs was captured in the transcriptome. However, the transcription activators of LBGs, *MYB90*, and *MYB1L* showed strong induction in C5. The *MYB90* can activate the promoters of UFGT, anthocyanidin 3-*O*-glucoside 6″-*O*-acyltransferase (3AT), and F3′5′H (Matus et al., 2017). In both genotypes, the abundance of *MYB90* was increased concomitantly with the abundance of *UFGT1*, although higher levels were detected in C5. It is documented that the MYB90/MYB1L acts downstream the AGL/ANL activators and the SPL repressor (Kobayashi et al., 2002; Walker et al., 2007; Xu et al., 2015). Concordantly, the expression levels of *SPL* sharply decreased during development, showing slight differences between genotypes. Interestingly, *AGL2* and *WD40* displayed higher levels during C5 berry development with the strongest signal detected in the seed of both genotypes. However, *AGL1* and *ANL2* contributed more during immature berry stages. One of the most interesting findings was the abundance of TFs encoded flavonoids/anthocyanin repressors, including the *MYB* and the *SLP* (Qian et al., 2017; Song et al., 2020). It could be speculated that the plenty of suppressor TFs in highly flavonoid accumulation genotypes will have a strong effect in adjusting the expression of downstream structural genes involved in the flavonoid pathway. Thus, such TF proteins can be present in plants and function as a backup system, avoiding the unnecessary excessive accumulation of flavonoids. Taken together, these results suggest that the modification reactions of flavonoids such as hydroxylation (*via* F3′H and F3′5′H), methylation (*via* FOMT), and glycosylation (*via* UFGT) are highly activated in C5, resulting in the production of more biologically active flavonoid derivatives.

## Data Availability Statement

The original contributions presented in the study are publicly available. This data can be found here: National Center for Biotechnology Information (NCBI) BioProject database under accession number PRJNA775666.

## Author Contributions

AI: conceptualization, methodology, validation, formal analysis, software, writing—original draft preparation, and visualization. AD: methodology, investigation, formal analysis, and writing—review and editing. MP: methodology, formal analysis, and software. PG: methodology, formal analysis, and investigation. VT and KS: conceptualization, and writing—review and editing. IE-S: conceptualization, methodology, validation, formal analysis, resources, data curation, writing—review and editing, visualization, supervision, project administration, and funding acquisition. All authors contributed to the article and approved the submitted version.

## Conflict of Interest

The authors declare that the research was conducted in the absence of any commercial or financial relationships that could be construed as a potential conflict of interest.

## Publisher’s Note

All claims expressed in this article are solely those of the authors and do not necessarily represent those of their affiliated organizations, or those of the publisher, the editors and the reviewers. Any product that may be evaluated in this article, or claim that may be made by its manufacturer, is not guaranteed or endorsed by the publisher.

## Footnotes

1
https://www.bioinformatics.babraham.ac.uk/projects/fastqc/

## References

[B1] Akashi T. Aoki T. Ayabe S. (2005). Molecular and biochemical characterization of 2-hydroxyisoflavanone dehydratase. Involvement of carboxylesterase-like proteins in leguminous isoflavone biosynthesis. *Plant Physiol.* 137 882–891. 10.1104/pp.104.056747 15734910 PMC1065389

[B2] Alam M. A. Subhan N. Hossain H. Hossain M. Reza H. M. Rahman M. M. (2016). Hydroxycinnamic acid derivatives: a potential class of natural compounds for the management of lipid metabolism and obesity. *Nutr. Metab.* 13:27. 10.1186/s12986-016-0080-3 27069498 PMC4827240

[B3] Andersen P. C. Sarkhosh A. Dustin H. Breman J. (2021). *The muscadine grape (Vitis rotundifolia Michx.). HS91.* Gainesville: University of Florida Institute of Food and Agricultural Sciences. 10.32473/edis-hs100-2020

[B4] Bai Y. Dougherty L. Xu K. (2014). Towards an improved apple reference transcriptome using RNA-seq. *Mol. Genet. Genom.* 289 427–438. 10.1007/s00438-014-0819-3 24532088

[B5] Barceló A. R. Pomar F. López-Serrano M. Pedreño M. A. (2003). Peroxidase: a multifunctional enzyme in grapevines. *Funct. Plant Biol.* 30 577–591. 10.1071/FP02096 32689044

[B6] Baudry A. Heim M. A. Dubreucq B. Caboche M. Weisshaar B. Lepiniec L. (2004). TT2, TT8, and TTG1 synergistically specify the expression of BANYULS and proanthocyanidin biosynthesis in *Arabidopsis thaliana*. *Plant J.* 39 366–380. 10.1111/j.1365-313X.2004.02138.x 15255866

[B7] Berdeja M. Nicolas P. Kappel C. Dai Z. W. Hilbert G. Peccoux A. (2015). Water limitation and rootstock genotype interact to alter grape berry metabolism through transcriptome reprogramming. *Hortic. Res.* 2:15012. 10.1038/hortres.2015.12 26504567 PMC4595978

[B8] Bindea G. Mlecnik B. Hackl H. Charoentong P. Tosolini M. Kirilovsky A. (2009). ClueGO: a Cytoscape plug-in to decipher functionally grouped gene ontology and pathway annotation networks. *Bioinformatics* 15 1091–1093. 10.1093/bioinformatics/btp101 19237447 PMC2666812

[B9] Bogs J. Downey M. O. Harvey J. S. Ashton A. R. Tanner G. J. Robinson S. P. (2005). Proanthocyanidin synthesis and expression of genes encoding leucoanthocyanidin reductase and anthocyanidin reductase in developing grape berries and grape vine leaves. *Plant Physiol. Biochem.* 139 652–663. 10.1104/pp.105.064238 16169968 PMC1255985

[B10] Bogs J. Jaffé F. W. Takos A. M. Walker A. R. Robinson S. P. (2007). The grapevine transcription factor VvMYBPA1 regulates proanthocyanidin synthesis during fruit development. *Plant Physiol.* 143 1347–1361. 10.1104/pp.106.093203 17208963 PMC1820911

[B11] Bolger A. M. Lohse M. Usadel B. (2014). Trimmomatic: a flexible trimmer for Illumina sequence data. *Bioinformatics* 30 2114–2120. 10.1093/bioinformatics/btu170 24695404 PMC4103590

[B12] Boss P. K. Davies C. Robinson S. P. (1996). Analysis of the expression of anthocyanin pathway genes in developing *Vitis vinifera* L. cv Shiraz grape berries and the implications for pathway regulation. *Plant Physiol.* 111 1059–1066. 10.1104/pp.111.4.1059 12226348 PMC160981

[B13] Boudet A. M. Kajita S. Grima-Pettenati J. Goffner D. (2003). Lignins and lignocellulosics: A better control of synthesis for new and improved uses. *Trends Plant Sci.* 8 576–581. 10.1016/j.tplants.2003.10.001 14659706

[B14] Bralley E. E. Hargrove J. L. Greenspan P. Hartle D. K. (2007). Topical anti-inflammatory activities of *Vitis rotundifolia* (muscadine grape) extracts in the tetradecanoyl phorbol acetate model of ear inflammation. *J. Med. Food* 10 636–642. 10.1089/jmf.2006.244 18158834

[B15] Chen Y. Lun A. T. L. Smyth G. K. (2016). From reads to genes to pathways: differential expression analysis of RNA-Seq experiments using Rsubread and the edgeR quasi-likelihood pipeline. *F1000Res* 5:1438. 10.12688/f1000research.8987.1 27508061 PMC4934518

[B16] Chong J. Baltz R. Schmitt C. Beffa R. Fritig B. Saindrenan P. (2002). Downregulation of a pathogen-responsive tobacco UDP-Glc: Phenylpropanoid Glucosyltransferase reduces scopoletin glycoside accumulation, enhances oxidative stress, and weakens virus resistance. *Plant Cell* 14 1093–1107. 10.1105/tpc.010436 12034899 PMC150609

[B17] Consortium G. O. (2004). The Gene Ontology (GO) database and informatics resource. *Nucleic Acids Res.* 32 D258–D261. 10.1093/nar/gkh036 14681407 PMC308770

[B18] Coombe B. G. McCarthy M. G. (2000). Dynamics of grape berry growth and physiology of ripening. *Aust. J. Grape Wine Res.* 6 131–135. 10.1111/j.1755-0238.2000.tb00171.x

[B19] Czemmel S. Heppel S. C. Bogs J. (2012). R2R3 MYB transcription factors: key regulators of the flavonoid biosynthetic pathway in grapevine. *Protoplasma* 249 109–118. 10.1007/s00709-012-0380-z 22307206

[B20] Czemmel S. Stracke R. Weisshaar B. Cordon N. Harris N. N. Walker A. R. (2009). The grapevine R2R3-MYB transcription factor VvMYBF1 regulates flavonol synthesis in developing grape berries. *Plant Physiol.* 151 1513–1530. 10.1104/pp.109.142059 19741049 PMC2773091

[B21] Dai Z. W. Léon C. Feil R. Lunn J. E. Delrot S. Gomès E. (2013). Metabolic profiling reveals coordinated switches in primary carbohydrate metabolism in grape berry (*Vitis vinifera* L.), a non-climacteric fleshy fruit. *J. Exp. Bot.* 64 1345–1355. 10.1093/jxb/ers396 23364938 PMC3598422

[B22] Darwish A. G. Das P. R. Ismail A. Gajjar P. Balasubramani S. P. Sheikh M. B. (2021). Untargeted metabolomics and antioxidant capacities of muscadine grape genotypes during berry development. *Antioxidants* 10:914. 10.3390/antiox10060914 34200012 PMC8230005

[B23] Degu A. Morcia C. Tumino G. Hochberg U. Toubiana D. Mattivi F. (2015). Metabolite profiling elucidates communalities and differences in the polyphenol biosynthetic pathways of red and white Muscat genotypes. *Plant Physiol. Biochem.* 86 24–33. 10.1016/j.plaphy.2014.11.006 25461697

[B24] Deluc L. G. Grimplet J. Wheatley M. D. Tillett R. L. Quilici D. R. Osborne C. (2007). Transcriptomic and metabolite analyses of Cabernet Sauvignon grape berry development. *BMC Genom.* 8:429. 10.1186/1471-2164-8-429 18034876 PMC2220006

[B25] Dixon R. A. (1999). “Isoflavonoids: biochemistry, molecular biology, and biological functions in: Comprehensive Natural Products Chemistry,” in *Polyketides and other Secondary Metabolites Including Fatty Acids and their Derivatives*, ed. Sankawa U. (Oxford: Elsevier), 773–823. 10.1016/B978-0-08-091283-7.00030-8

[B26] Du H. Huang Y. Tang Y. (2010). Genetic and metabolic engineering of isoflavonoid biosynthesis. *Appl. Microbiol. Biotechnol.* 86 1293–1312. 10.1007/s00253-010-2512-8 20309543

[B27] Duan S. Wu Y. Fu R. Wang L. Chen Y. Xu W. (2019). Comparative metabolic profiling of grape skin tissue along grapevine berry developmental stages reveals systematic influences of root restriction on skin metabolome. *Int. J. Mol. Sci.* 20:534. 10.3390/ijms20030534 30695987 PMC6386830

[B28] Dubos C. Le Gourrierec J. Baudry A. Huep G. Lanet E. Debeaujon I. (2008). MYBL2 is a new regulator of flavonoid biosynthesis in *Arabidopsis thaliana*. *Plant J.* 55 940–953. 10.1111/j.1365-313X.2008.03564.x 18532978

[B29] Fasoli M. Dal Santo S. Zenoni S. Tornielli G. B. Farina L. Zamboni A. (2012). The grapevine expression atlas reveals a deep transcriptome shift driving the entire plant into a maturation program. *Plant Cell* 24 3489–3505. 10.1105/tpc.112.100230 22948079 PMC3480284

[B30] Ferreyra M. L. F. Rius S. P. Casati P. (2012). Flavonoids: biosynthesis, biological functions, and biotechnological applications. *Front. Plant Sci.* 3:222. 10.3389/fpls.2012.00222 23060891 PMC3460232

[B31] Fraissinet-Tachet L. Baltz R. Chong J. Kauffmann S. Fritig B. Saindrenan P. (1998). Two tobacco genes induced by infection, elicitor and salicylic acid encode glucosyltransferases acting on phenylpropanoids and benzoic acid derivatives, including salicylic acid. *FEBS Lett.* 437 319–323. 10.1016/S0014-5793(98)01257-5 9824316

[B32] Gagné S. Lacampagne S. Claisse O. Gény L. (2009). Leucoanthocyanidin reductase and anthocyanidin reductase gene expression and activity in flowers, young berries and skins of Vitis vinifera L. cv. Cabernet-Sauvignon during development. *Plant Physiol. Biochem.* 47 282–290. 10.1016/j.plaphy.2008.12.004 19136268

[B33] Gambino G. Perrone I. Gribaudo I. (2008). A rapid and effective method for RNA extraction from different tissues of grapevine and other woody plants. *Phytochem. Anal.* 19 520–525. 10.1002/pca.1078 18618437

[B34] García-Gómez B. E. Ruiz D. Salazar J. A. Rubio M. Martínez-García P. J. Martínez-Gómez P. (2020). Analysis of metabolites and gene expression changes relative to apricot (*Prunus armeniaca* L.) fruit quality during development and ripening. *Front. Plant Sci.* 11:1269. 10.3389/fpls.2020.01269 32973833 PMC7466674

[B35] Giménez E. Dominguez E. Pineda B. Heredia A. Moreno V. Lozano R. (2015). Transcriptional activity of the MADS box ARLEQUIN/TOMATO AGAMOUS-LIKE1 gene is required for cuticle development of tomato fruit. *Plant Physiol.* 168 1036–1048. 10.1104/pp.15.00469 26019301 PMC4741332

[B36] God J. M. Tate P. Larcom L. L. (2007). Anticancer effects of four varieties of muscadine grape. *J. Med. Food* 10 54–59. 10.1089/jmf.2006.699 17472467

[B37] Gomez-Plaza E. Gil-Munoz R. Lopez-Roca J. M. Martinez-Cutillas A. Fernandez-Fernandez J. I. (2001). Phenolic compounds and color stability of red wines: Effect of skin maceration time. *Am. J. Enol. Vitic.* 52 266–270.

[B38] Gonzalez A. Zhao M. Leavitt J. M. Lloyd A. M. (2008). Regulation of the anthocyanin biosynthetic pathway by the TTG1/bHLH/Myb transcriptional complex in *Arabidopsis* seedlings. *Plant J.* 53 814–827. 10.1111/j.1365-313X.2007.03373.x 18036197

[B39] Gou J. Y. Felippes F. F. Liu C. J. Weigel D. Wang J. W. (2011). Negative regulation of anthocyanin biosynthesis in *Arabidopsis* by a miR156-targeted SPL transcription factor. *Plant Cell* 23 1512–1522. 10.1105/tpc.111.084525 21487097 PMC3101539

[B40] Gourineni V. Shay N. F. Chung S. Sandhu A. K. Gu L. (2012). Muscadine grape (*Vitis rotundifolia*) and wine phytochemicals prevented obesity-associated metabolic complications in C57BL/6J mice. *J. Agric. Food Chem.* 60 7674–7681. 10.1021/jf3013663 22788667

[B41] He X. Szewczyk P. Karyakin A. Evin M. Hong W.-X. Zhang Q. (2010). Structure of a cation-bound multidrug and toxic compound extrusion transporter. *Nature* 467 991–994. 10.1038/nature09408 20861838 PMC3152480

[B42] Hsu Y. H. Tagami T. Matsunaga K. Okuyama M. Suzuki T. Noda N. (2017). Functional characterization of UDP-rhamnose-dependent rhamnosyltransferase involved in anthocyanin modification, a key enzyme determining blue coloration in Lobelia erinus. *Plant J.* 89 325–337. 10.1111/tpj.13387 27696560

[B43] Hunt L. Holdsworth M. J. Gray J. E. (2007). Nicotinamidase activity is important for germination. *Plant J.* 51 341–351. 10.1111/j.1365-313X.2007.03151.x 17587307

[B44] Ibrahim R. K. Bruneau A. Bantignies B. (1998). Plant O-methyltransferase: molecular analysis, common signature, and classification. *Plant Mol. Biol.* 36 1–10. 10.1023/A:1005939803300 9484457

[B45] Jaillon O. Aury J. M. Noel B. Policriti A. Clepet C. Casagrande A. (2007). The grapevine genome sequence suggests ancestral hexaploidization in major angiosperm phyla. *Nature* 449 463–467. 10.1038/nature06148 17721507

[B46] Jeong S. T. Goto-Yamamoto N. Hashizume K. Esaka M. (2006). Expression of the flavonoid 3′-hydroxylase and flavonoid 3′,5′-hydroxylase genes and flavonoid composition in grape (*Vitis vinifera*). *Plant Sci.* 170 61–69. 10.1016/j.plantsci.2005.07.025

[B47] Kim B. G. Sung S. H. Chong Y. Lim Y. Ahn J. H. (2010). Plant flavonoid O-methyltransferases: Substrate specificity and application. *J. Plant Biol.* 53 321–329. 10.1007/s12374-010-9126-7

[B48] Kobayashi S. Ishimaru M. Hiraoka K. Honda C. (2002). Myb-related genes of the Kyoho grape (*Vitis labrusca*) regulate anthocyanin biosynthesis. *Planta* 215 924–933. 10.1007/s00425-002-0830-5 12355152

[B49] Kubo H. Peeters A. J. M. Aarts M. G. M. Pereira A. Koornneef M. (1999). ANTHOCYANINLESS2, a homeobox gene affecting anthocyanin distribution and root development in *Arabidopsis*. *Plant Cell* 11 1217–1226. 10.1105/tpc.11.7.1217 10402424 PMC144283

[B50] Langfelder P. Horvath S. (2008). WGCNA: an R package for weighted correlation network analysis. *BMC Bioinform.* 9:559. 10.1186/1471-2105-9-559 19114008 PMC2631488

[B51] Li H. Li J. Dong Y. Hao H. Ling Z. Bai H. (2019). Time-series transcriptome provides insights into the gene regulation network involved in the volatile terpenoid metabolism during the flower development of lavender. *BMC Plant Biol.* 19:313. 10.1186/s12870-019-1908-6 31307374 PMC6632208

[B52] Li S. (2014). Transcriptional control of flavonoid biosynthesis: fine-tuning of the MYB-bHLH-WD40 (MBW) complex. *Plant Signal Behav.* 9:e27522. 10.4161/psb.27522 24393776 PMC4091223

[B53] Li S. T. Zachgo S. (2013). TCP3 interacts with R2R3-MYB proteins, promotes flavonoid biosynthesis and negatively regulates the auxin response in *Arabidopsis thaliana*. *Plant J.* 76 901–913. 10.1111/tpj.12348 24118612

[B54] Love M. I. Huber W. Anders S. (2014). Moderated estimation of fold change and dispersion for RNA-seq data with DESeq2. *Genome Biol.* 15:550. 10.1186/s13059-014-0550-8 25516281 PMC4302049

[B55] Luo J. Song S. Wei Z. Huang Y. Zhang Y. Lu J. (2017). The comparative study among different fractions of muscadine grape ‘Noble’ pomace extracts regarding anti-oxidative activities, cell cycle arrest, and apoptosis in breast cancer. *Food Nutr. Res.* 61:1412795. 10.1080/16546628.2017.1412795 29249924 PMC5727431

[B56] Massonnet M. Fasoli M. Tornielli G. B. Altieri M. Sandri M. Zuccolotto P. (2017). Ripening transcriptomic program in red and white grapevine varieties correlates with berry skin anthocyanin accumulation. *Plant Physiol.* 174 2376–2396. 10.1104/pp.17.00311 28652263 PMC5543946

[B57] Matsui K. Umemura Y. Ohme-Takagi M. (2008). AtMYBL2, a protein with a single MYB domain, acts as a negative regulator of anthocyanin biosynthesis in *Arabidopsis*. *Plant J.* 55 954–967. 10.1111/j.1365-313X.2008.03565.x 18532977

[B58] Matus J. T. Cavallini E. Loyola R. Höll J. Finezzo L. Dal Santo S. (2017). A group of grapevine MYBA transcription factors located in chromosome 14 control anthocyanin synthesis in vegetative organs with different specificities compared with the berry color locus. *Plant J.* 91 220–236. 10.1111/tpj.13558 28370629

[B59] Mellen P. B. Daniel K. R. Brosnihan K. B. Hansen K. J. Herrington D. M. (2010). Effect of muscadine grape seed supplementation on vascular function in subjects with or at risk for cardiovascular disease: a randomized crossover trial. *J. Am. Coll. Nutr.* 29 469–475. 10.1080/07315724.2010.10719883 21504973 PMC3313487

[B60] Mendonca P. Darwish A. G. Tsolova V. El-Sharkawy I. Soliman K. F. A. (2019). The anticancer and antioxidant effects of muscadine grape extracts on racially different triple-negative breast cancer cells. *Anticancer Res.* 39 4043–4053. 10.21873/anticanres.13560 31366486 PMC7754981

[B61] Merdinoglu D. Wiedeman-Merdinoglu S. Coste P. Dumas V. Haetty S. Butterlin G. (2003). Genetic analysis of downy mildew resistance derived from *Muscadinia rotundifolia*. *Acta Hortic.* 603 451–456. 10.17660/ActaHortic.2003.603.57 34854763

[B62] Nakamura T. Ichinose H. Wariishi H. (2012). Flavin-containing monooxygenases from *Phanerochaete chrysosporium* responsible for fungal metabolism of phenolic compounds. *Biodegradation* 23 343–350. 10.1007/s10532-011-9521-x 22102096

[B63] Nwafor C. C. Gribaudo I. Schneider A. Wehrens R. Grando M. S. Costantini L. (2014). Transcriptome analysis during berry development provides insights into co-regulated and altered gene expression between a seeded wine grape variety and its seedless somatic variant. *BMC Genom.* 15:1030. 10.1186/1471-2164-15-1030 25431125 PMC4301461

[B64] Olien W. C. (1990). The muscadine grape: botany, viticulture, history, and current industry. *HortScience* 25 732–739. 10.21273/HORTSCI.25.7.732

[B65] Oliveros J. C. (2007). *Venny. An interactive tool for comparing lists with Venn diagrams.* Available online at: https://bioinfogp.cnb.csic.es/tools/venny/index.html.

[B66] Palumbo M. C. Zenoni S. Fasoli M. Massonnet M. Farina L. Castiglione F. (2014). Integrated network analysis identifies fight-club nodes as a class of hubs encompassing key putative switch genes that induce major transcriptome reprogramming during grapevine development. *Plant Cell* 26 4617–4635. 10.1105/tpc.114.133710 25490918 PMC4311215

[B67] Park M. Vera D. Kambiranda D. Gajjar P. Cadle-Davidson L. Tsolova V. (2021). Chromosome-level genome sequence assembly and genome-wide association study of *Muscadinia rotundifolia* reveal the genetics of 12 berry-related traits. *Hortic. Res.* 9:uhab011. 10.1093/hr/uhab011 35040982 PMC8769032

[B68] Pastrana-Bonilla E. Akoh C. C. Sellappan S. Krewer G. (2003). Phenolic content and antioxidant capacity of muscadine grapes. *J. Agric. Food Chem.* 51 5497–5503. 10.1021/jf030113c 12926904

[B69] Patro R. Duggal G. Love M. I. Irizarry R. A. Kingsford C. (2017). Salmon: fast and bias-aware quantification of transcript expression using dual-phase inference. *Nat. Methods* 14 417–419. 10.1038/nmeth.4197 28263959 PMC5600148

[B70] Pinu F. R. (2018). Grape and wine metabolomics to develop new insights using untargeted and targeted approaches. *Ferment* 4:92. 10.3390/fermentation4040092

[B71] Prasain J. K. Barnes S. (2014). “Polyphenols in chronic diseases and their mechanisms of action,” in *polyphenols in human health and disease*, eds Watson R. S. Preedy V. R. Zibadi S. (Florida, FL: Academic Press), 1401–1419.

[B72] Qian M. Ni J. Niu Q. Bai S. Bao L. Li J. (2017). Response of miR156-SPL module during the red peel coloration of bagging-treated Chinese sand pear (*Pyrus pyrifolia* Nakai). *Front. Physiol.* 8:550. 10.3389/fphys.2017.00550 28824447 PMC5545762

[B73] Randhir R. Lin Y. T. Shetty K. (2004). Stimulation of phenolics, antioxidant and antimicrobial activities in dark germinated mung bean sprouts in response to peptide and phytochemical elicitors. *Process Biochem.* 39 637–646. 10.1016/S0032-9592(03)00197-3 15331344

[B74] Raudvere U. Kolberg L. Kuzmin I. Arak T. Adler P. Peterson H. (2019). g:Profiler: a web server for functional enrichment analysis and conversions of gene lists (2019 update). *Nucleic Acids Res.* 47 W191–W198. 10.1093/nar/gkz369 31066453 PMC6602461

[B75] Rippert P. Puyaubert J. Grisollet D. Derrier L. Matringe M. (2009). Tyrosine and phenylalanine are synthesized within the plastids in *Arabidopsis*. *Plant Physiol.* 149 1251–1260. 10.1104/pp.108.130070 19136569 PMC2649395

[B76] Šikuten I. Štambuk P. Andabaka Ž Tomaz I. Marković Z. Stupić D. (2020). Grapevine as a rich source of polyphenolic compounds. *Molecules* 25:5604. 10.3390/molecules25235604 33260583 PMC7731206

[B77] Song L. Wang X. Han W. Qu Y. Wang Z. Zhai R. (2020). PbMYB120 negatively regulates anthocyanin accumulation in pear. *Int. J. Mol. Sci.* 21:1528. 10.3390/ijms21041528 32102306 PMC7073189

[B78] Staudt G. (1997). Evaluation of resistance to grapevine powdery mildew (*Uncinula necator* Schw. Burr., anamorph *Oidium tuckeri* Berk.) in accessions of *Vitis* species. *Vitis* 36 151–154.

[B79] Stracke R. Ishihara H. Huep G. Barsch A. Mehrtens F. Niehaus K. (2007). Differential regulation of closely related R2R3-MYB transcription factors controls flavonol accumulation in different parts of the *Arabidopsis thaliana* seedling. *Plant J.* 50 660–677. 10.1111/j.1365-313X.2007.03078.x 17419845 PMC1976380

[B80] Sweetman C. Wong D. C. Ford C. M. Drew D. P. (2012). Transcriptome analysis at four developmental stages of grape berry (*Vitis vinifera* cv. Shiraz) provides insights into regulated and coordinated gene expression. *BMC Genom.* 13:691. 10.1186/1471-2164-13-691 23227855 PMC3545830

[B81] Walker A. Lee E. Bogs J. McDavid D. Thomas M. Robinson S. (2007). White grapes arose through the mutation of two similar and adjacent regulatory genes. *Plant J.* 49 772–785. 10.1111/j.1365-313X.2006.02997.x 17316172

[B82] Wang J. De Luca V. (2005). The biosynthesis and regulation of biosynthesis of Concord grape fruit esters, including ‘foxy’ methylanthranilate. *Plant J.* 44 606–619. 10.1111/j.1365-313X.2005.02552.x 16262710

[B83] Wang L. Li Y. Jin X. Liu L. Dai X. Liu Y. (2020). Floral transcriptomes reveal gene networks in pineapple floral growth and fruit development. *Commun. Biol.* 3:500. 10.1038/s42003-020-01235-2 32913289 PMC7483743

[B84] Wang Y. S. Xu Y. J. Gao L. P. Yu O. Wang X. Z. He X. J. (2014). Functional analysis of flavonoid 3′,5′-hydroxylase from tea plant (*Camellia sinensis*): critical role in the accumulation of catechins. *BMC Plant Biol.* 14:347. 10.1186/s12870-014-0347-7 25490984 PMC4275960

[B85] Widhalm J. R. Dudareva N. (2015). A familiar ring to it: Biosynthesis of plant benzoic acids. *Mol. Plant* 8 83–97. 10.1016/j.molp.2014.12.001 25578274

[B86] Winkel-Shirley B. (2001). Flavonoid biosynthesis: a colorful model for genetics, biochemistry, cell biology and biotechnology. *Plant Physiol.* 126 485–493. 10.1104/pp.126.2.485 11402179 PMC1540115

[B87] Xu W. Dubos C. Lepiniec L. (2015). Transcriptional control of flavonoid biosynthesis by MYB-bHLH-WDR complexes. *Trends Plant Sci.* 20 176–185. 10.1016/j.tplants.2014.12.001 25577424

[B88] Yan H. Pei X. Zhang H. Li X. Zhang X. Zhao M. (2021). MYB-mediated regulation of anthocyanin biosynthesis. *Int. J. Mol. Sci.* 22:3103. 10.3390/ijms22063103 33803587 PMC8002911

[B89] You Q. Chen F. Sharp J. L. Wang X. You Y. Zhang C. (2012). High-performance liquid chromatography–mass spectrometry and evaporative light-scattering detector to compare phenolic profiles of muscadine grapes. *J. Chromatogr. A* 1240 96–103. 10.1016/j.chroma.2012.03.086 22520637

[B90] Zimmermann I. Heim M. Weisshaar B. Uhrig J. (2004). Comprehensive identification of *Arabidopsis thaliana* MYB transcription factors interacting with R/B-like bHLH proteins. *Plant J.* 40 22–34. 10.1111/j.1365-313X.2004.02183.x 15361138

