# Supplementary material for: Transcriptome Profiling During Muscadine Berry Development Reveals the Dynamic of Polyphenols Metabolism
Source: Front Plant Sci. 2022 Feb 2;12:818071. doi: 10.3389/fpls.2021.818071 (PMC8849228; doi:10.3389/fpls.2021.818071)
Supplement: Supplementary file 1 [file Data_Sheet_1.PDF]

Transcriptome profiling during muscadine berry development reveals the dynamic of polyphenols metabolism

Ahmed Ismail[1,2], Ahmed G. Darwish[1,3], Minkyu Park[1], Pranavkumar Gajjar[1], Violeta Tsolova[1], Karam F. A. Soliman[4], Islam El-Sharkawy[1*].

This supplemental file contains the supplementary figures S1 – S24.

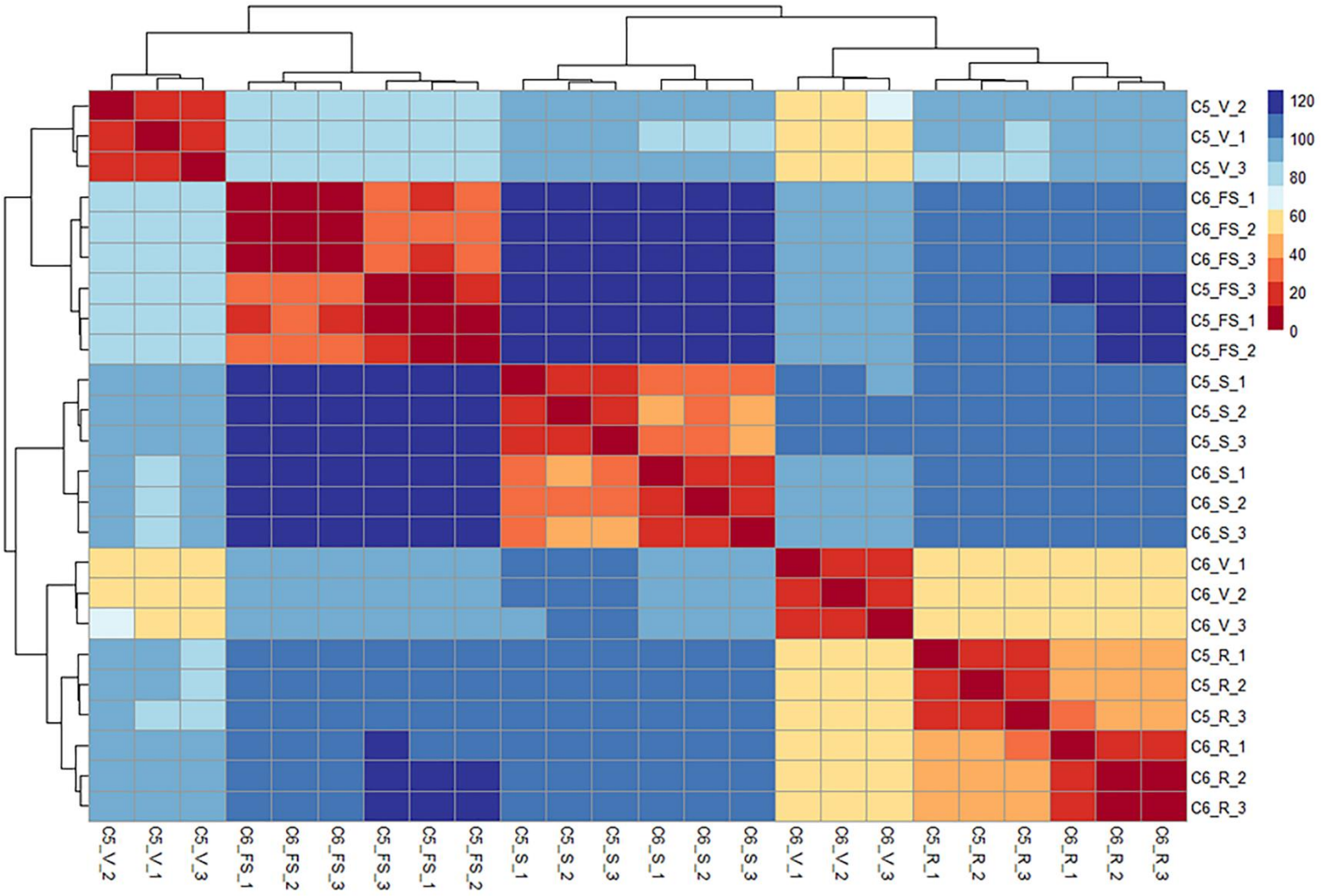

Fig S1. Heatmap of sample-to-sample distances of RNA-seq data from berries at different developmental stages of the two muscadine genotypes (C5 and C6) using the variance stabilizing transformation (VST) and Euclidean distances. The developmental stages are as follows: fruit-set (FS), véraison (V), ripe skin/flesh (R), and ripe seeds (S).

Transcriptome-Associated Antioxidant Profiling

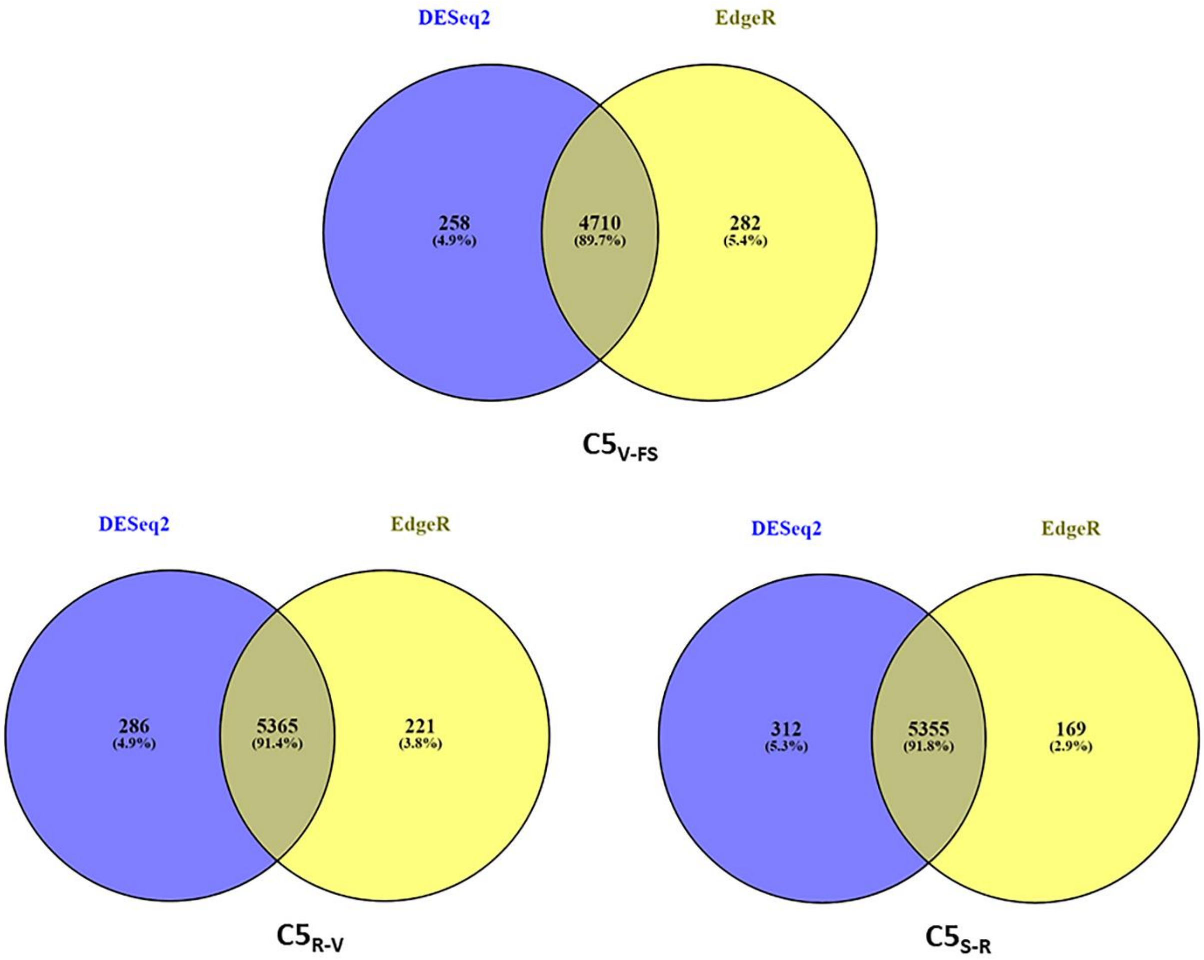

Fig. S2. Venn diagrams of DEGs in the C5 muscadine genotype. Genes that are differentially expressed when each time point is compared to its earlier point in C5 genotype, using DESeq2 or EdgeR pipelines resulted in non-redundant 10116 genes. The log2fold change of those genes are > 1.5 or < –1.5. Fruit-set (FS), véraison (V), ripe skin/flesh (R), and ripe seeds (S).

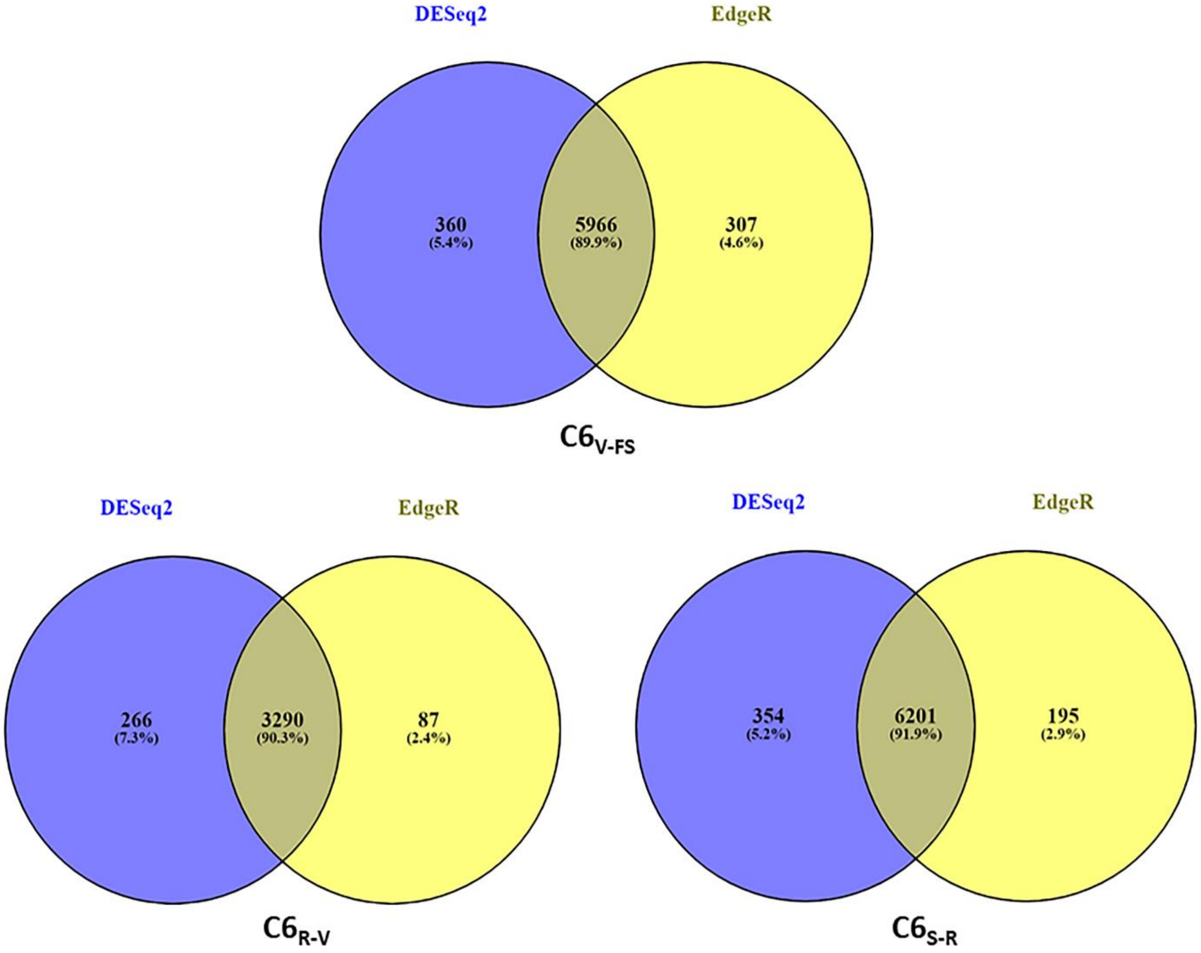

Fig. S3. Venn diagrams of DEGs in C6 muscadine genotype. Genes that are differentially expressed when each time point is compared to its earlier point in C6 genotype, using DESeq2 or EdgeR pipelines resulted in non-redundant 10543 genes. The log2fold change of those genes are > 1.5 or < –1.5. Fruit-set (FS), véraison (V), ripe skin/flesh (R), and ripe seeds (S).

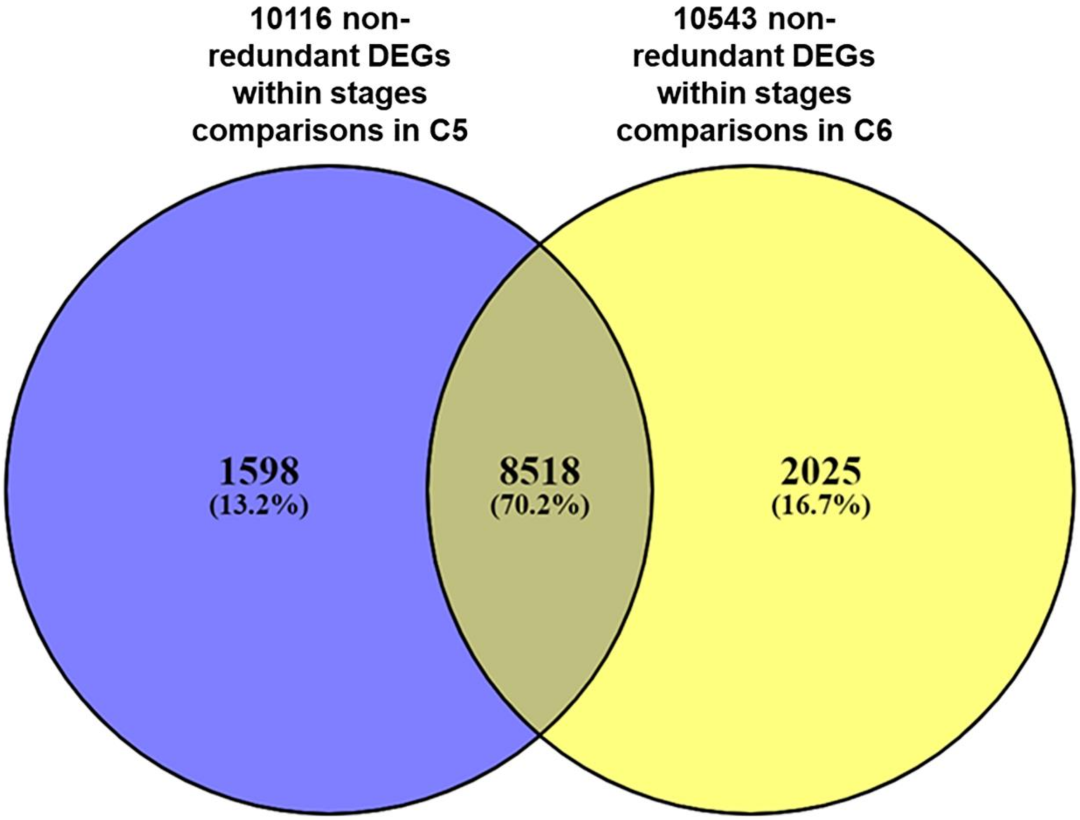

Fig. S4. Venn diagrams of DEGs when stages in muscadine C5 or C6 were compared against its former one within the same genotype generated by DESeq2 and EdgeR pipelines, as well as they are distinct. The log2fold change of those genes are > 1.5 or < –1.5. The resultant number of differentially genes were 10116 or 10543 in C5 or C6, respectively. Fruit-set (FS), véraison (V), ripe skin/flesh (R), and ripe seeds (S).

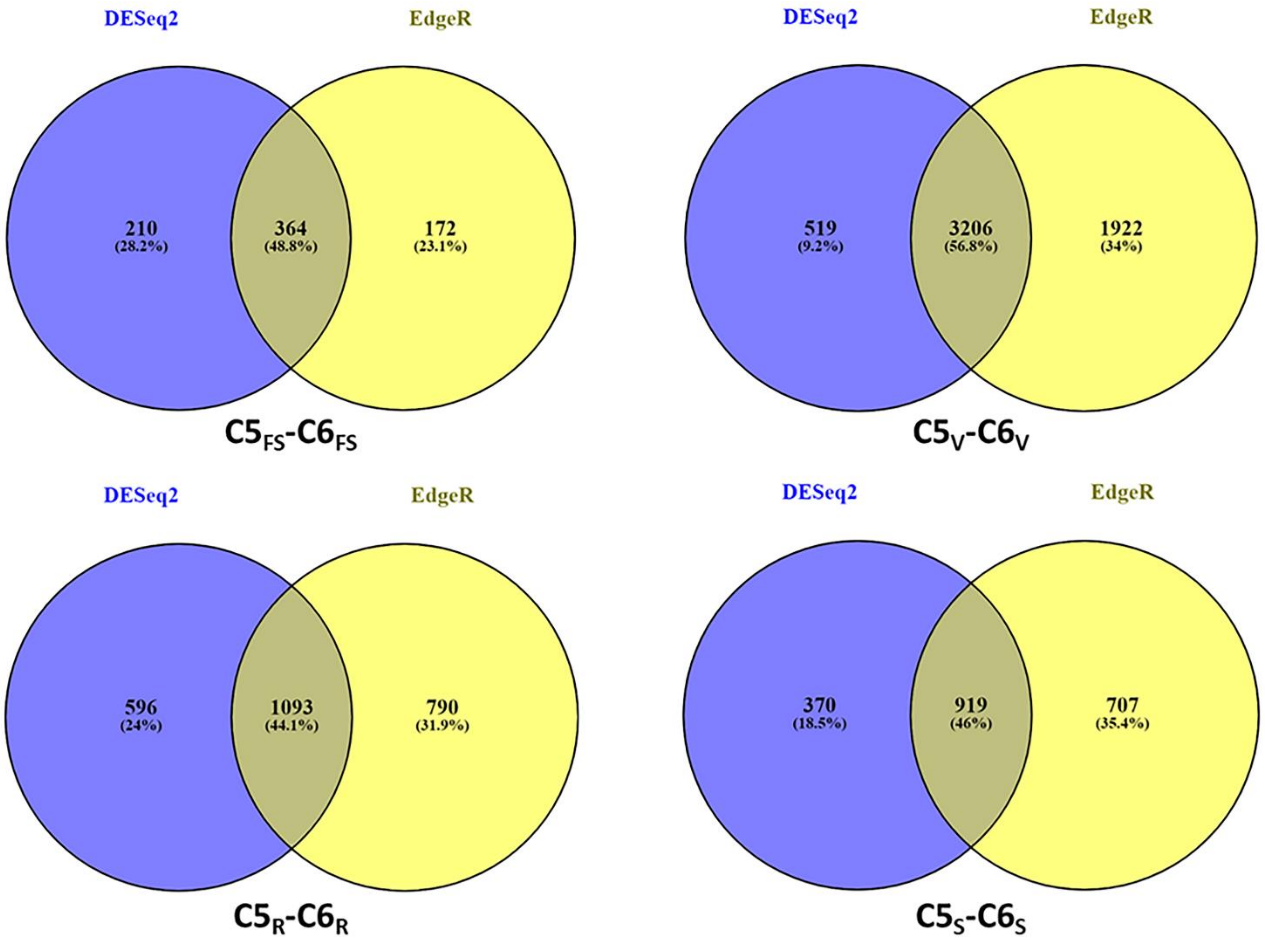

Fig. S5. Venn diagrams of DEGs when muscadine C5 stages were compared to its corresponding C6 stages, using DESeq2 or EdgeR pipelines resulted in non-redundant 7772 genes. The log2fold change of those genes are > 1.5 or < –1.5. Fruit-set (FS), véraison (V), ripe skin/flesh (R), and ripe seeds (S).

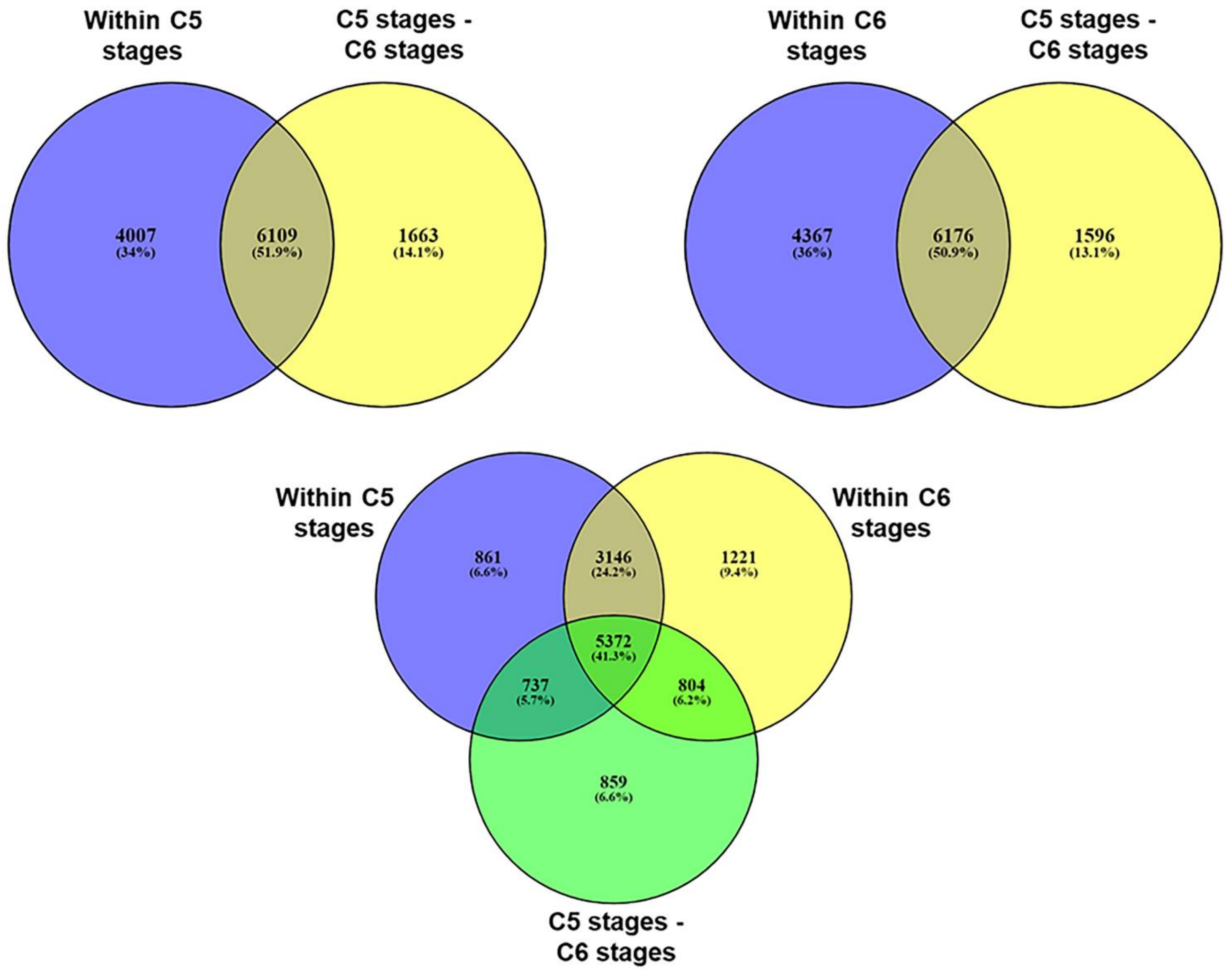

Fig. S6. Venn diagrams of DEGs when stages in muscadine C5 or C6 were compared against its earlier one within the same genotype or stage in C5 was compared against its corresponding stage in C6 using DESeq2 and EdgeR pipelines. The log2fold change of those genes are > 1.5 or < –1.5. The resultant number of DEGs were 10116, 10543, or 7772 in C5, C6, or C5 stage against its corresponding C6, respectively.

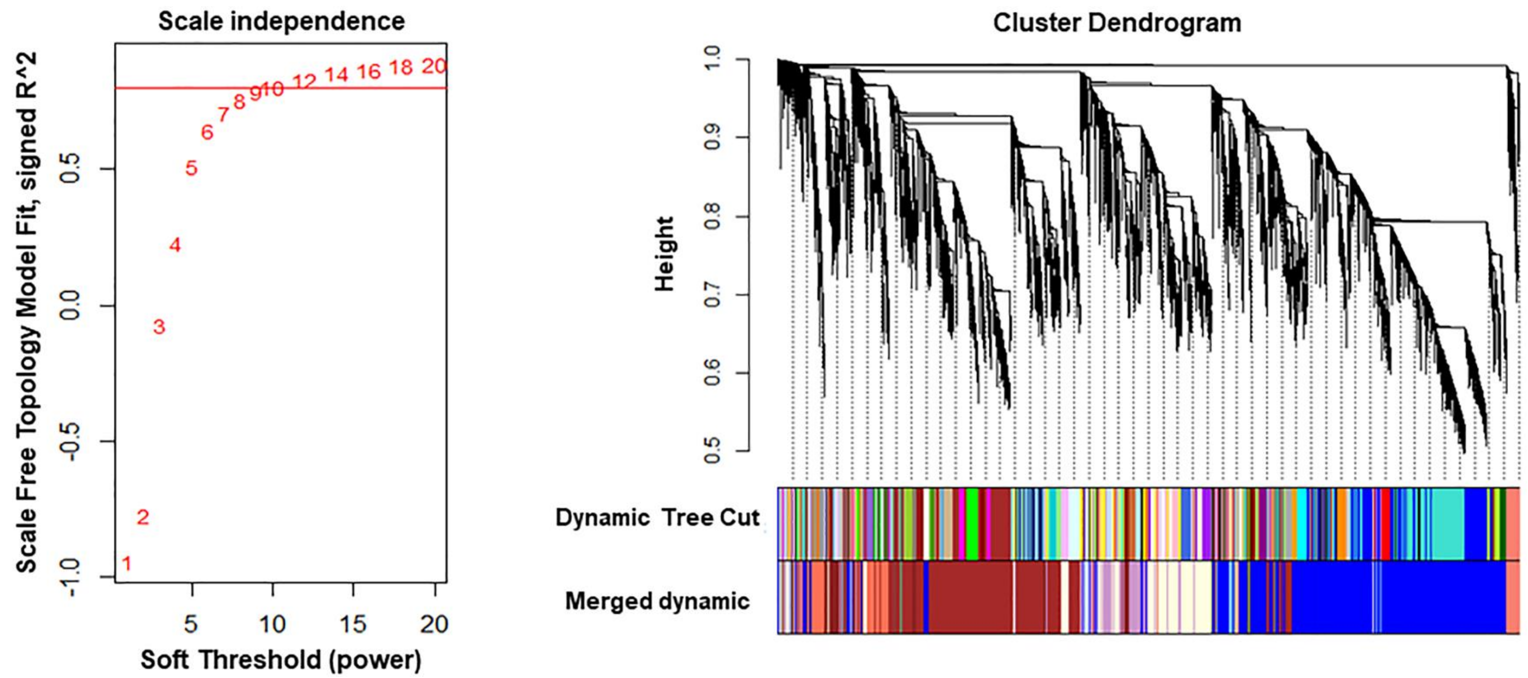

Fig. S7. (A) Analysis of network topology to determinate the soft thresholding power for module construction. As shown, the power 10 is the best soft threshold power for this dataset. (B) Hierarchical cluster dendrogram of (20886) genes showing co-expressed modules identified by weighted gene co-expression network analysis for the muscadine (C5 and C6 genotype) RNA-seq data. Each leaf on the tree is counted for one gene. The color row underneath the dendrogram shows the 16 merged module colors (based on a threshold of 0.25), as well as the original module colors, labeled with different colors.

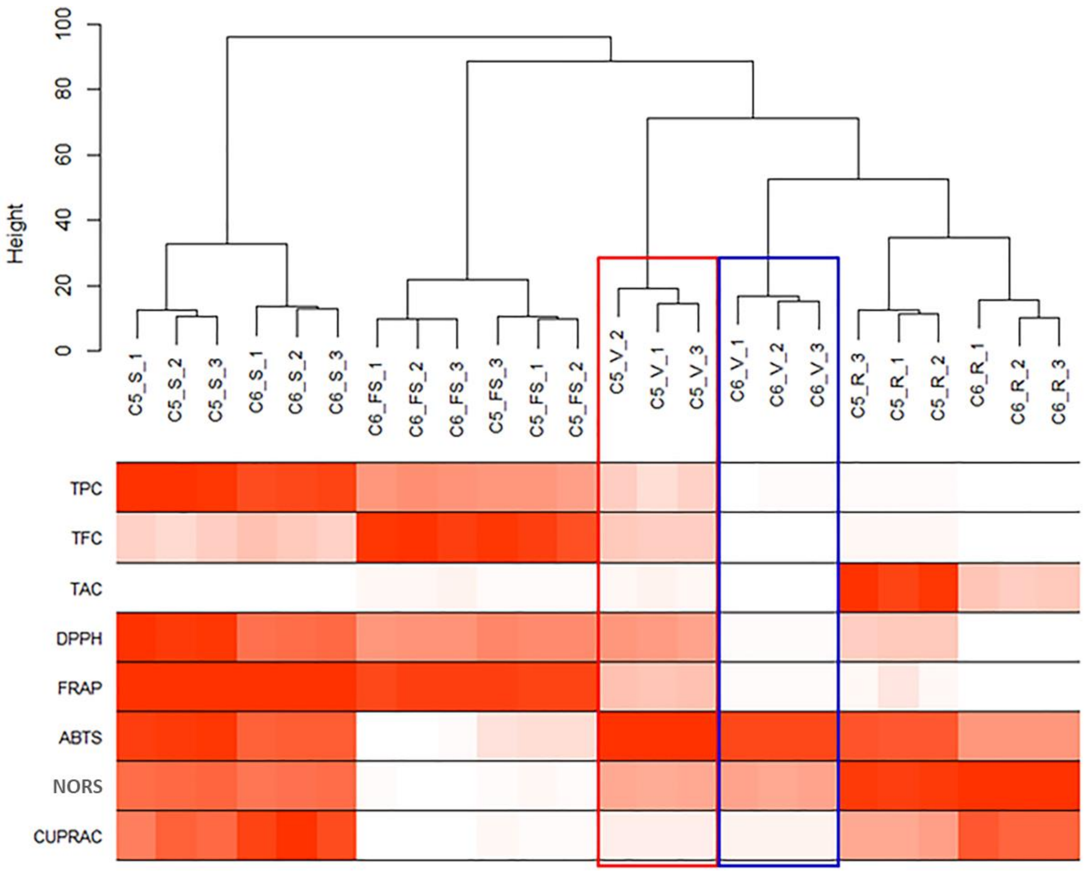

Fig. S8. Clustering/sample dendrogram and trait heatmap of samples based on their Euclidean distance. Clustering dendrogram of 24 RNA-seq samples from muscadine berry during developmental stages based on their Euclidean distance. Trait heatmap shows the association of samples with biochemical-related traits, including total phenolic content (TPC), total flavonoid content (TFC), total anthocyanin content (TAC), and different types of antioxidant assays (DPPH, FRAP, ABTS, NORS, and CUPRAC) from C5 and C6 genotypes at different berry developmental stages. Red and white color represents high and low values, respectively. The véraison stage of the C5 and C6, showing high diversity are indicated by red- and blue-color rectangle, respectively.

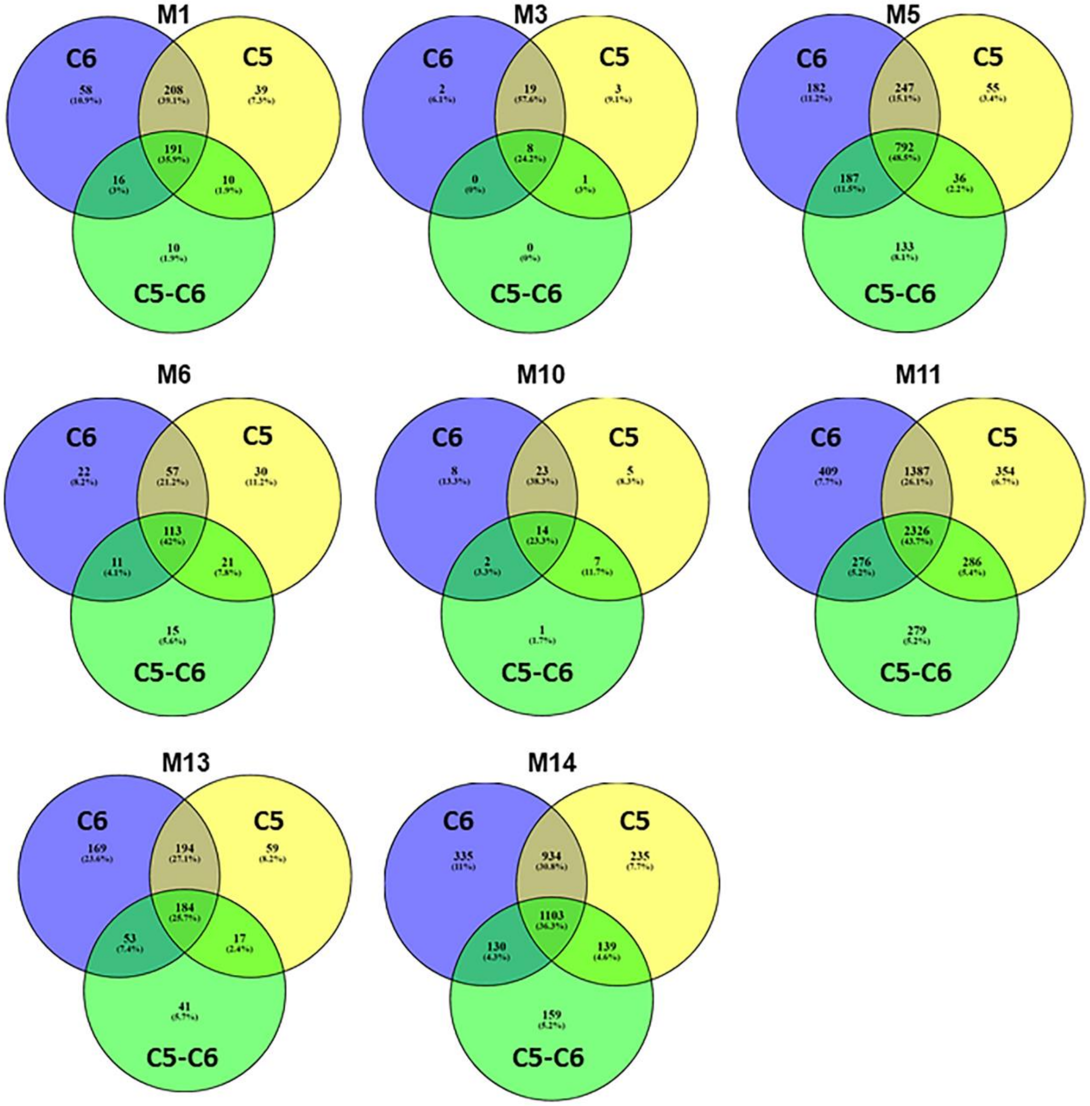

Fig. S9. Venn diagrams of DEGs from the three comparisons ($C5_{stages}$, $C6_{stages}$, and $C5_{stage}$ - $C6_{stage}$) and their association in WGCNA module of interest.

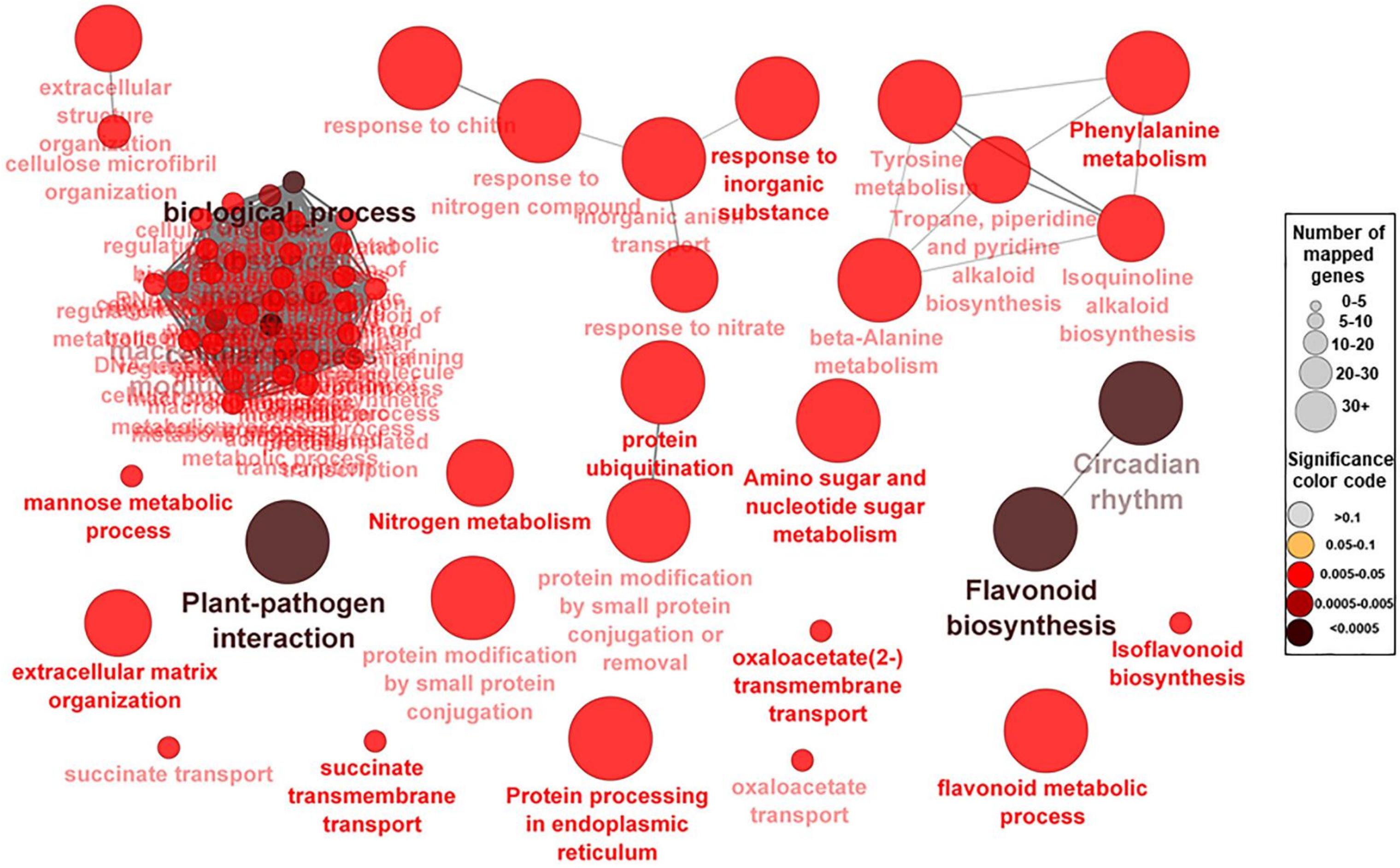

Fig. S10. A network view for the predefined Biological processes GO terms and KEEG that are overrepresented in the cluster WGCNA module ME1 (*p* adjusted <0.05), extracted by g:Profiler website with Benjamini-Hochberg FDR multiple testing correction method. The default ClueGO settings were applied, and the terms are functionally grouped based on shared genes (kappa score). The size of the nodes indicates the number of mapped genes, while the color indicates the degree of significance (0.1< pValue <0.0005). The most significant term defines the name of the group.

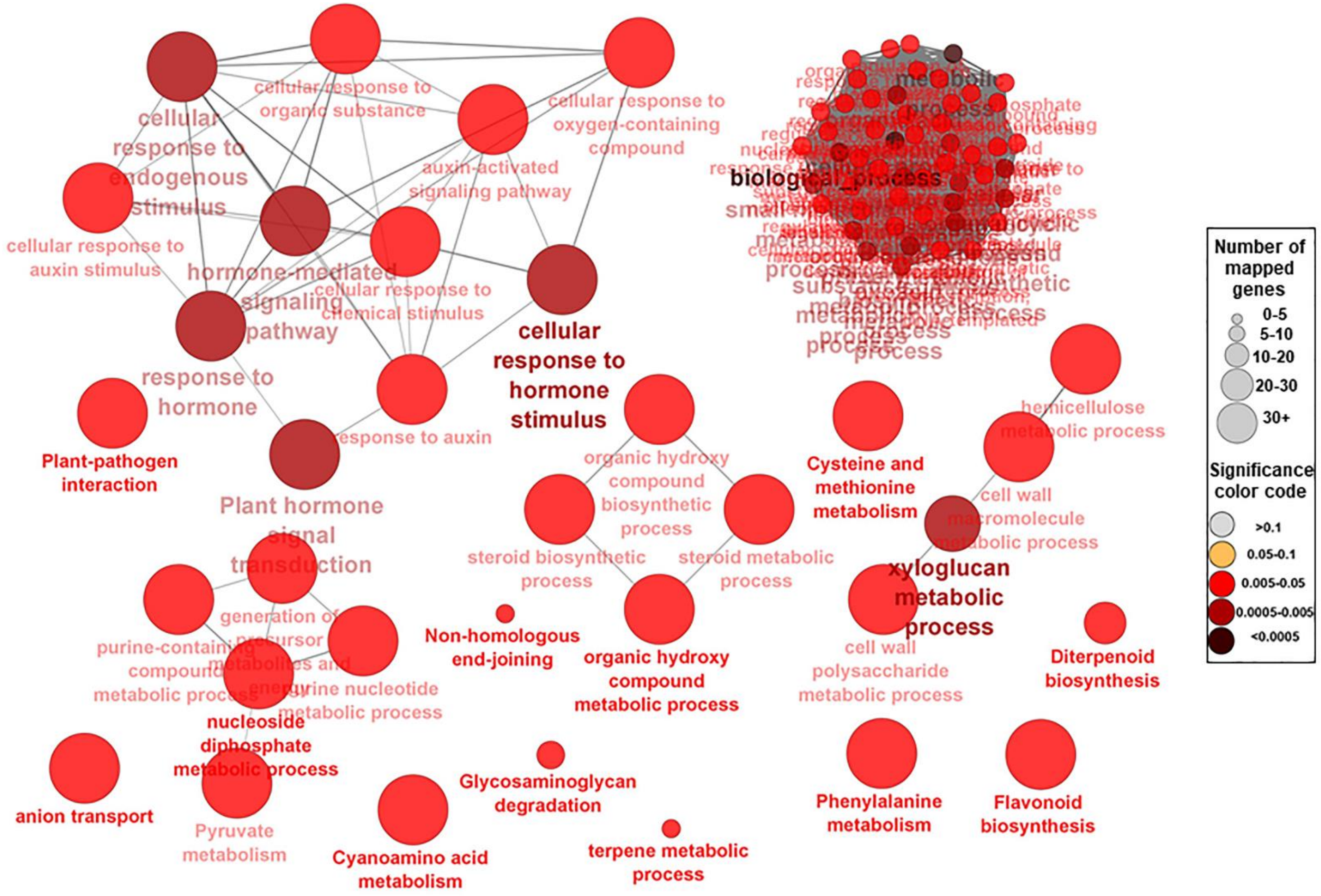

Fig. S11. A network view for the predefined Biological processes GO terms and KEEG that are overrepresented in the cluster WGCNA module ME5 (p adjusted <0.05), extracted by g:Profiler website with Benjamini-Hochberg FDR multiple testing correction method. The default ClueGO settings were applied, and the terms are functionally grouped based on shared genes (kappa score). The size of the nodes indicates the number of mapped genes, while the color indicates the degree of significance (0.1< pValue <0.0005). The most significant term defines the name of the group.

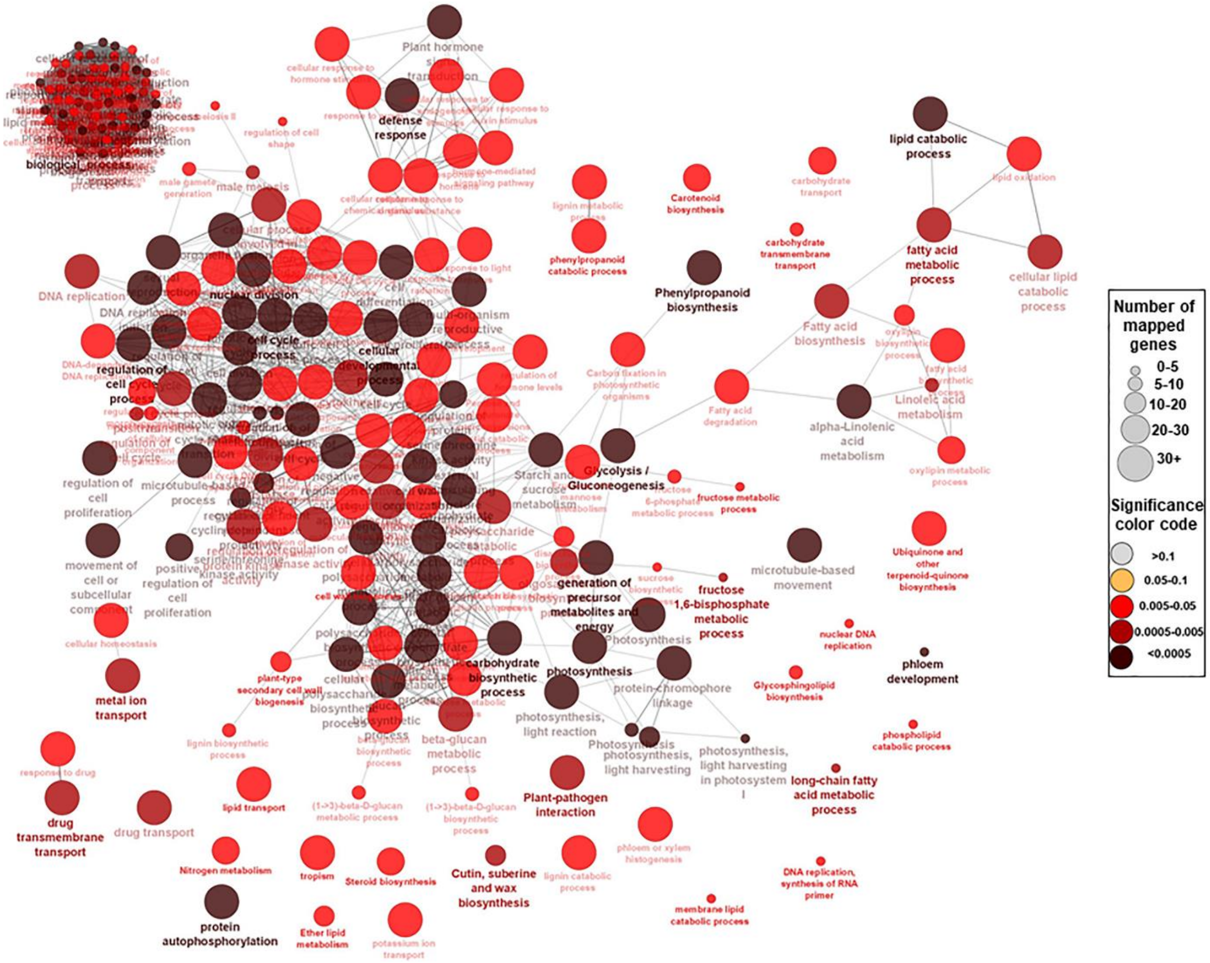

Fig. S12. A network view for the predefined Biological processes GO terms and KEEG that are overrepresented in the cluster WGCNA module ME11 (p adjusted <0.05), extracted by g:Profiler website with Benjamini-Hochberg FDR multiple testing correction method. The default ClueGO settings were applied, and the terms are functionally grouped based on shared genes (kappa score). The size of the nodes indicates the number of mapped genes, while the color indicates the degree of significance (0.1< pValue <0.0005). The most significant term defines the name of the group.

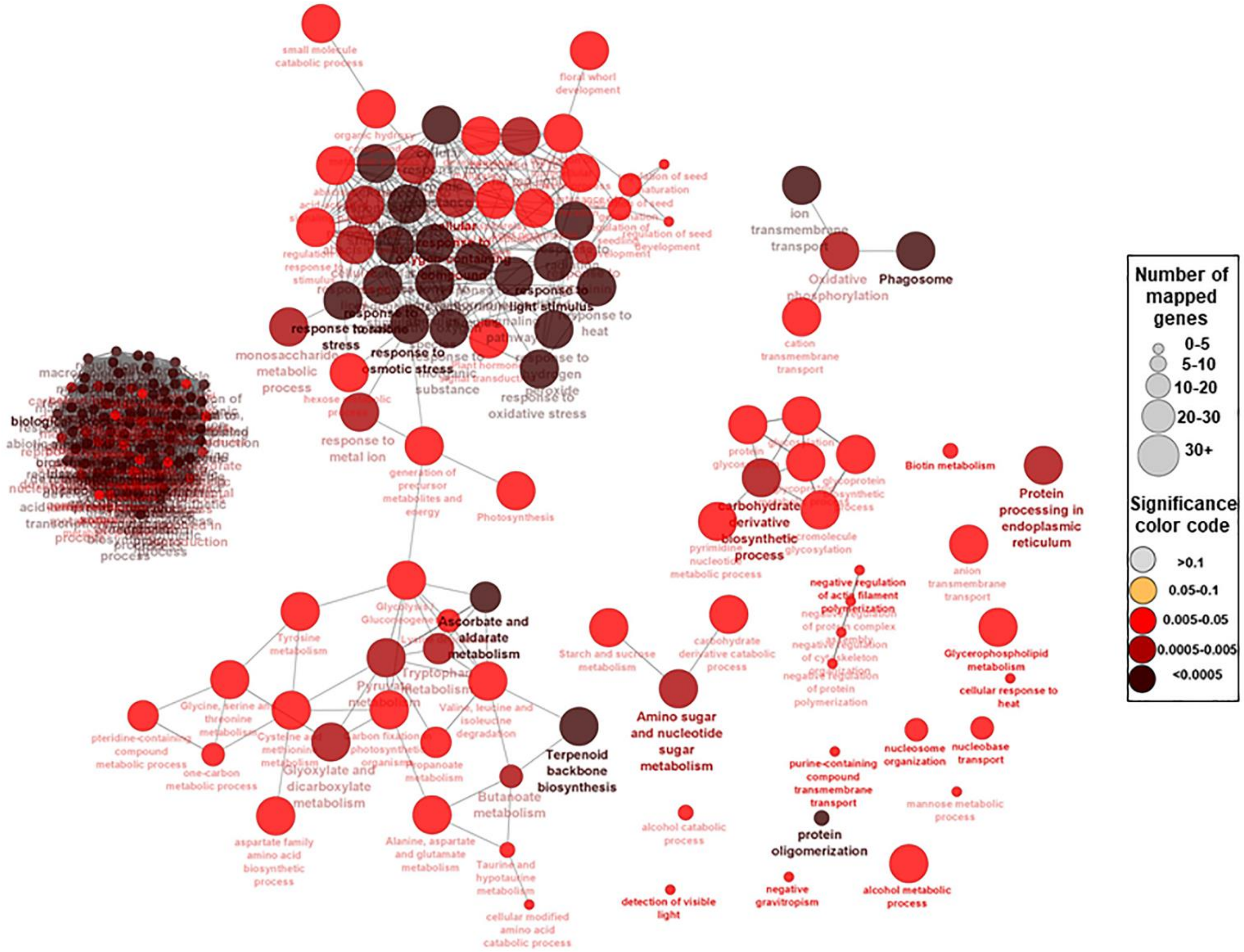

Fig. S13. A network view for the predefined Biological processes GO terms and KEEG that are overrepresented in the cluster WGCNA module ME14 (p adjusted <0.05), extracted by g:Profiler website with Benjamini-Hochberg FDR multiple testing correction method. The default ClueGO settings were applied, and the terms are functionally grouped based on

shared genes (kappa score). The size of the nodes indicates the number of mapped genes, while the color indicates the degree of significance (0.1< pValue <0.0005). The most significant term defines the name of the group.

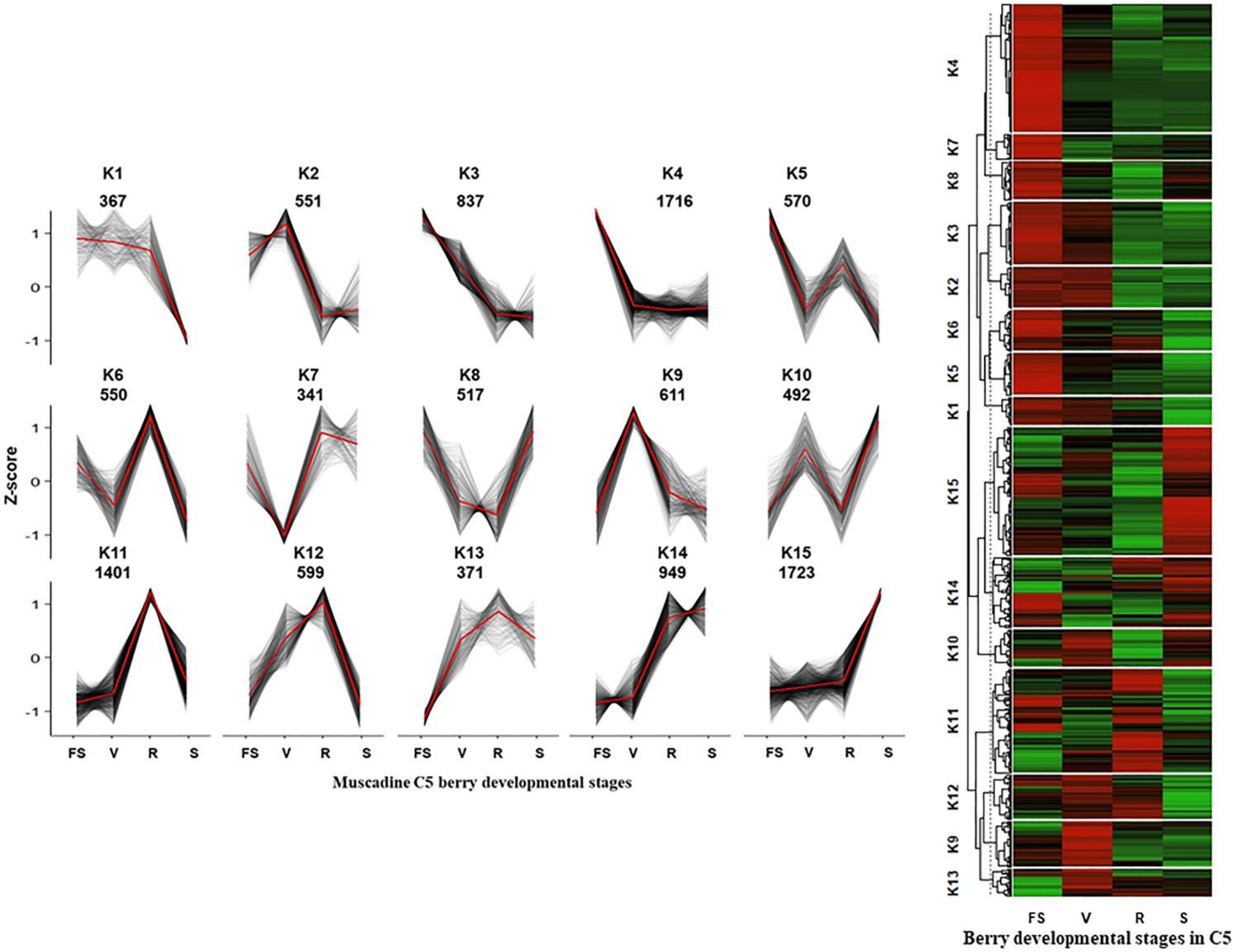

Fig. S14. K-means clustering of 11595 non-redundant DEGs represented in WGCNA modules of interest in C5 genotype. By using transcript per million (TPM) of genes in C5, K-means algorithm generated 15 clusters (K1 to K15) of 11595 non-redundant DEGs represented in WGCNA modules of interest (ME1, 3, 5, 6, 10, 11, 13, and 14). A number of 11591 out of

11595 non-redundant DEGs are depicted in the heatmap of K-mean clusters. Fruit-set (FS), véraison (V), ripe skin/flesh (R), and ripe seeds (S).

Transcriptome-Associated Antioxidant Profiling

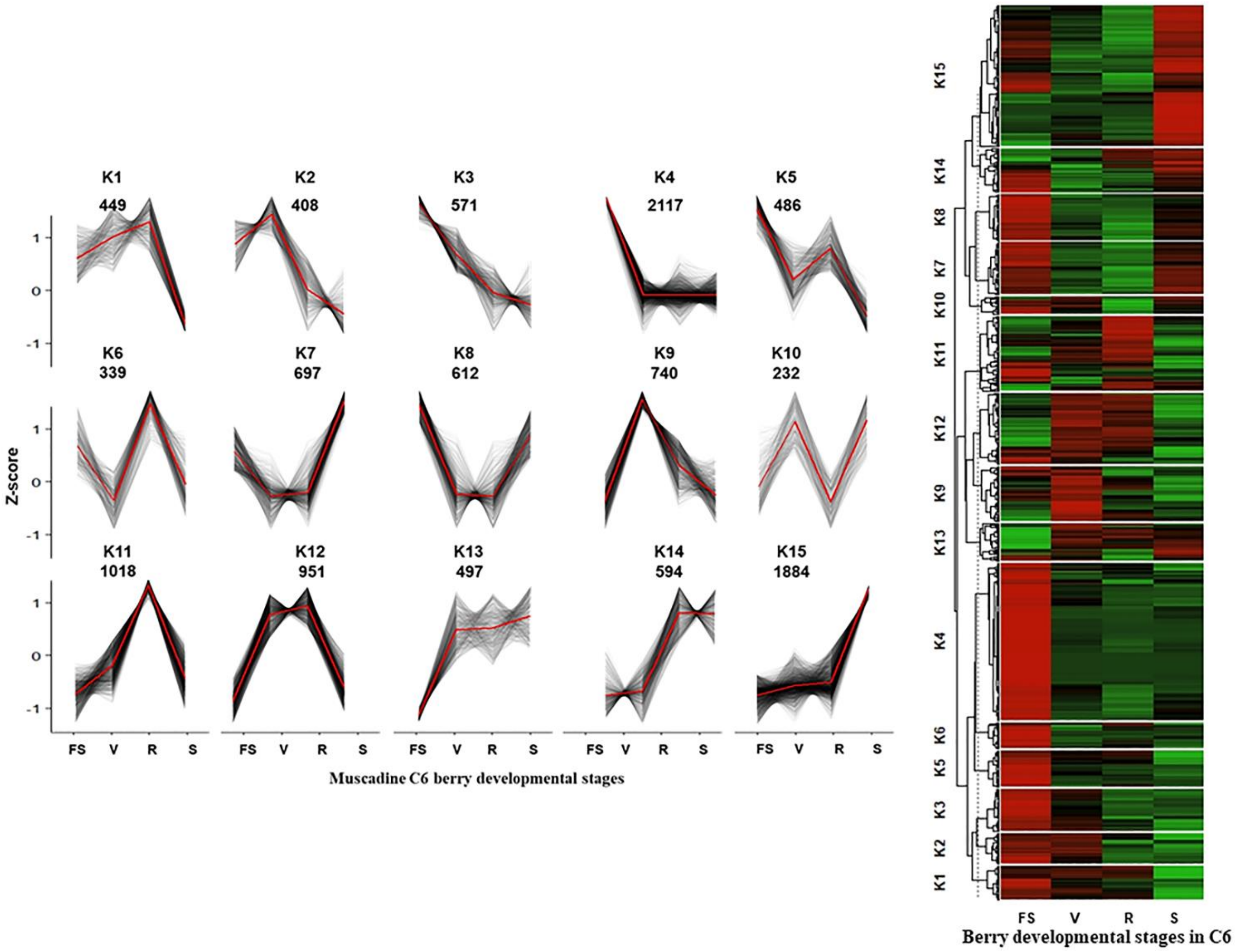

Fig. S15. K-means clustering and heatmap of 11595 non-redundant DEGs represented in WGCNA modules of interest in C6 genotype: By using transcript per million (TPM) of genes in C6, Kmeans algorithm generated 15 clusters (K1 to K15) of 11595 non-redundant DEGs represented in WGCNA modules of interest (ME1, 3, 5, 6, 10, 11, 13, and 14). A number of

11584 out of 11595 non-redundant DEGs are depicted in the heatmap of K-mean clusters. Fruit-set (FS), véraison (V), ripe skin/flesh (R), and ripe seeds (S).

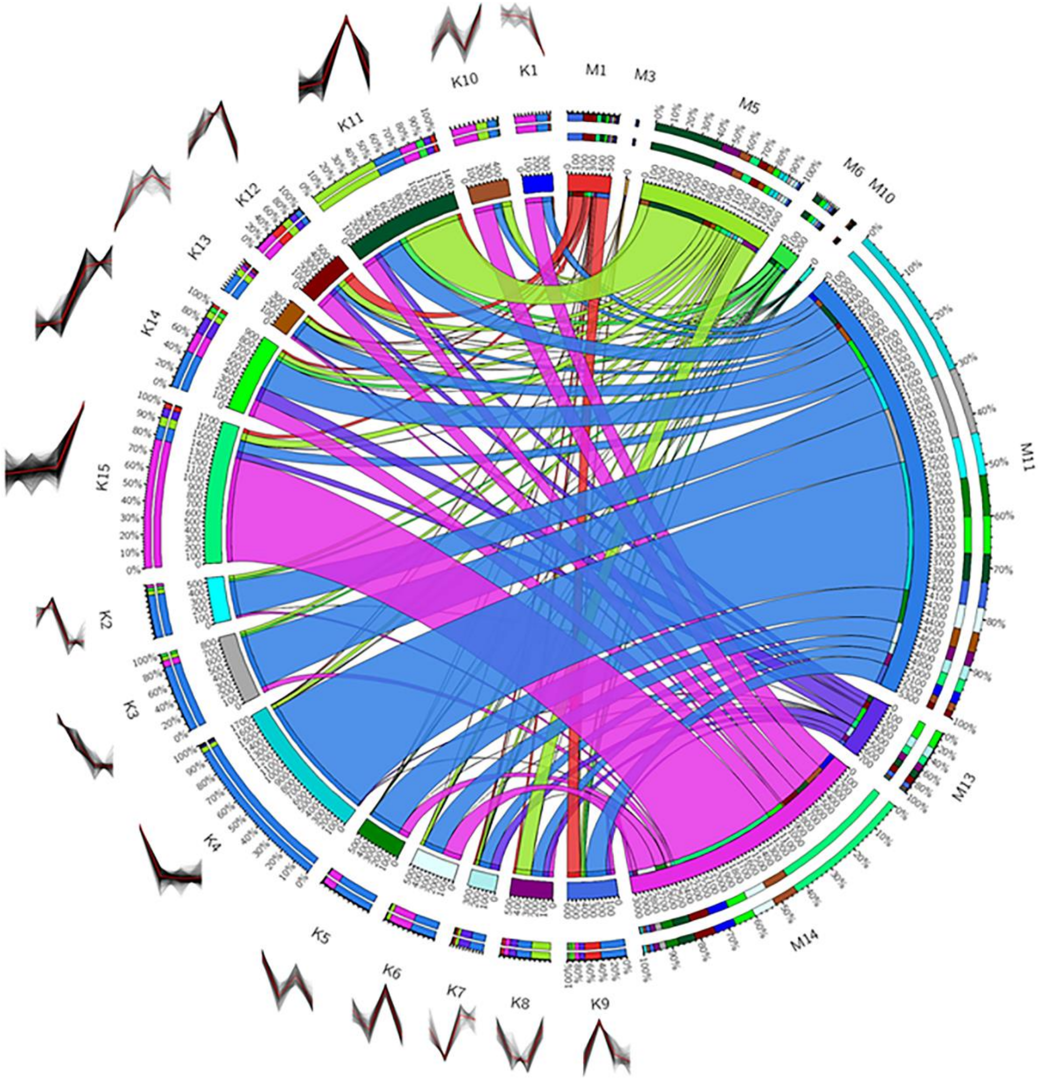
K10
K1
M1
M3
M5
M6
M10
M11
M13
M14
K9
K8
K7
K6
K5
K4
K3
K2
K15
K14
K13
K12
K11

Fig. S16. Circos of the 15 K-mean clusters of 11595 non-redundant DEGs represented in WGCNA modules of interest and C5 genotype. The 15 K-mean clusters represented in muscadine C5 genotype (11595 non-redundant genes) against the 8 WGCNA modules of interest.

Transcriptome-Associated Antioxidant Profiling

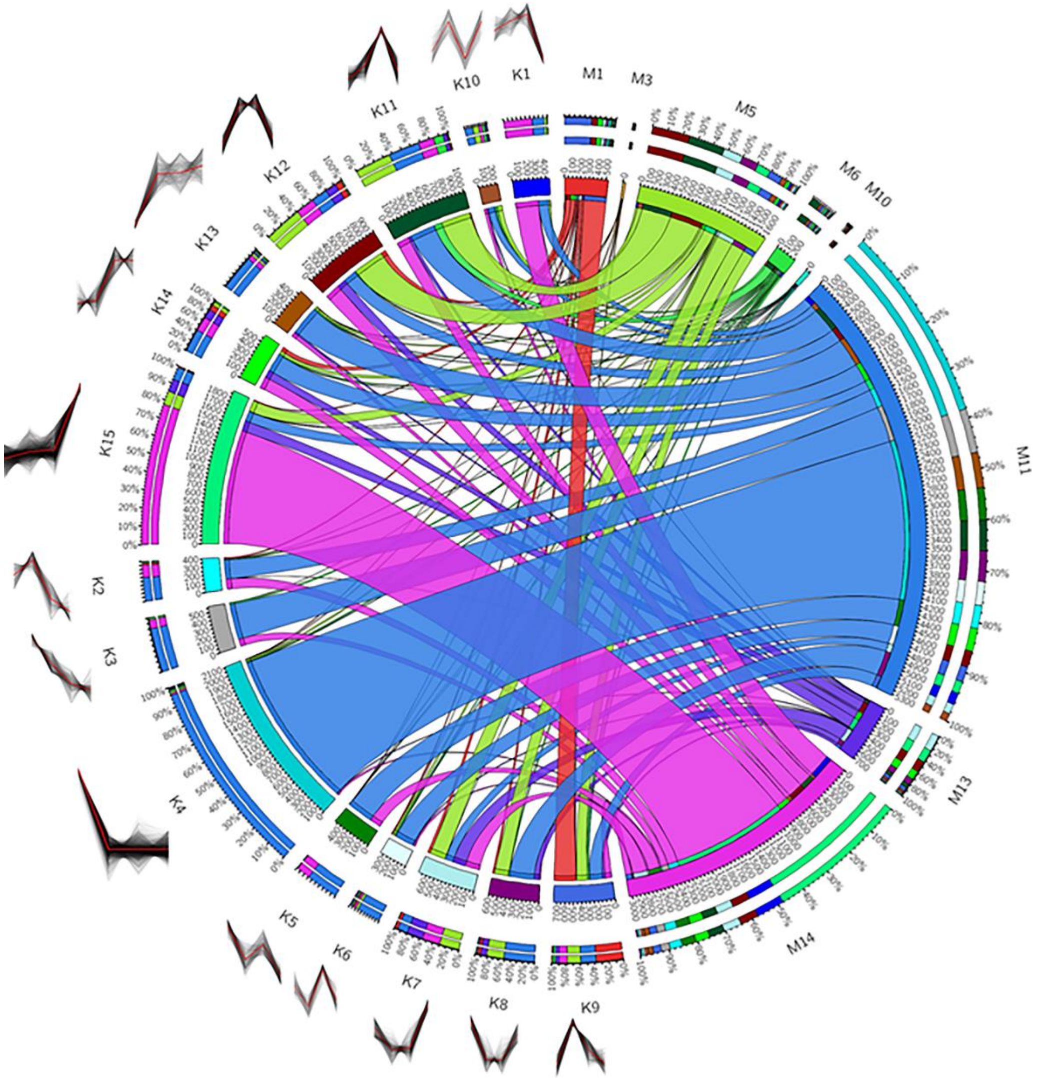

Fig. S17. Circos of the 15 K-mean clusters of 11595 non-redundant DEGs represented in WGCNA modules of interest and C6 genotype. The 15 K-mean clusters represented in muscadine C6 genotype (11595 non-redundant genes) against the 8 WGCNA modules of interest.

Transcriptome-Associated Antioxidant Profiling

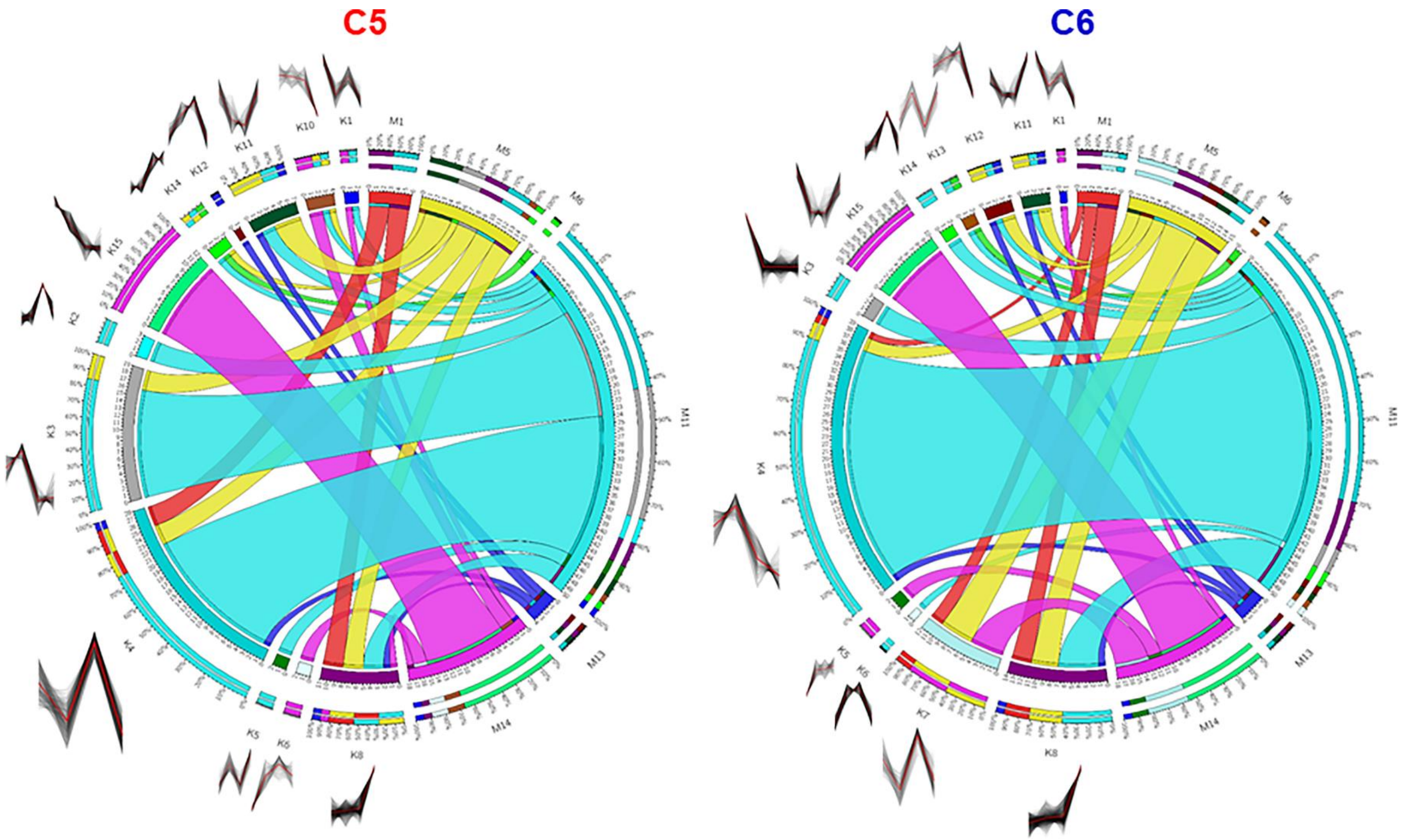

Fig. S18. Circos of the 15 K-mean clusters of the selected 94 DEGs represented in the 6 WGCNA modules of C5 and C6 genotypes. A 12 out of 15 K-mean clusters represented in muscadine C5 and C6 genotypes against the 6 WGCNA modules.

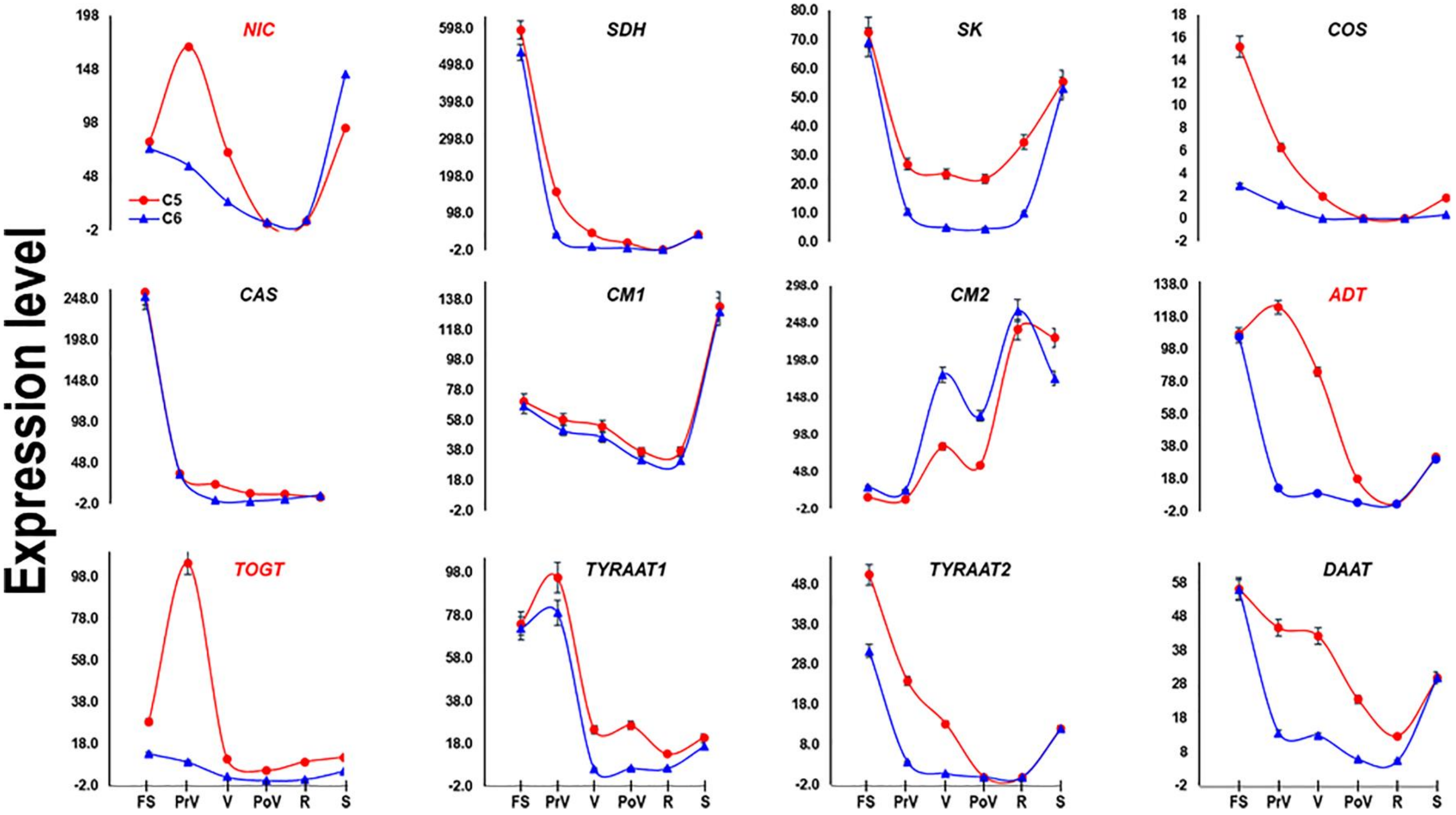

Fig. S19. Shikimic & Amino Acids Pathway. The curves show the expression pattern of genes involved in shikimic and amino acids pathway that were assessed by qPCR at different berry developmental stages, including fruit-set (FS), pre-véraison (PrV), véraison (V), post-véraison (PoV), ripe skin/flesh (R), and ripe seeds (S). *NIC*, nicotinamidase; *SDH*, shikimate dehydrogenase; *SK*, shikimate kinase; *COS*, chorismate synthase; *CAS*, caffeine synthase; *CM*, chorismate mutase; *ADT*, arogenate dehydratase/prephenate dehydratase; *TOGT*, tyrosine biosynthetic process; *TYRAAT*, arogenate dehydrogenase; *DAAT*, *D*-amino-acid transaminase. Red-colored gene names represent the transcripts exhibiting different

patterns, where the gene was significantly expressed in C5 at specific stages. Black-colored gene names represent the transcripts showing similar patterns with different kinetics/levels between C5 and C6 genotypes.

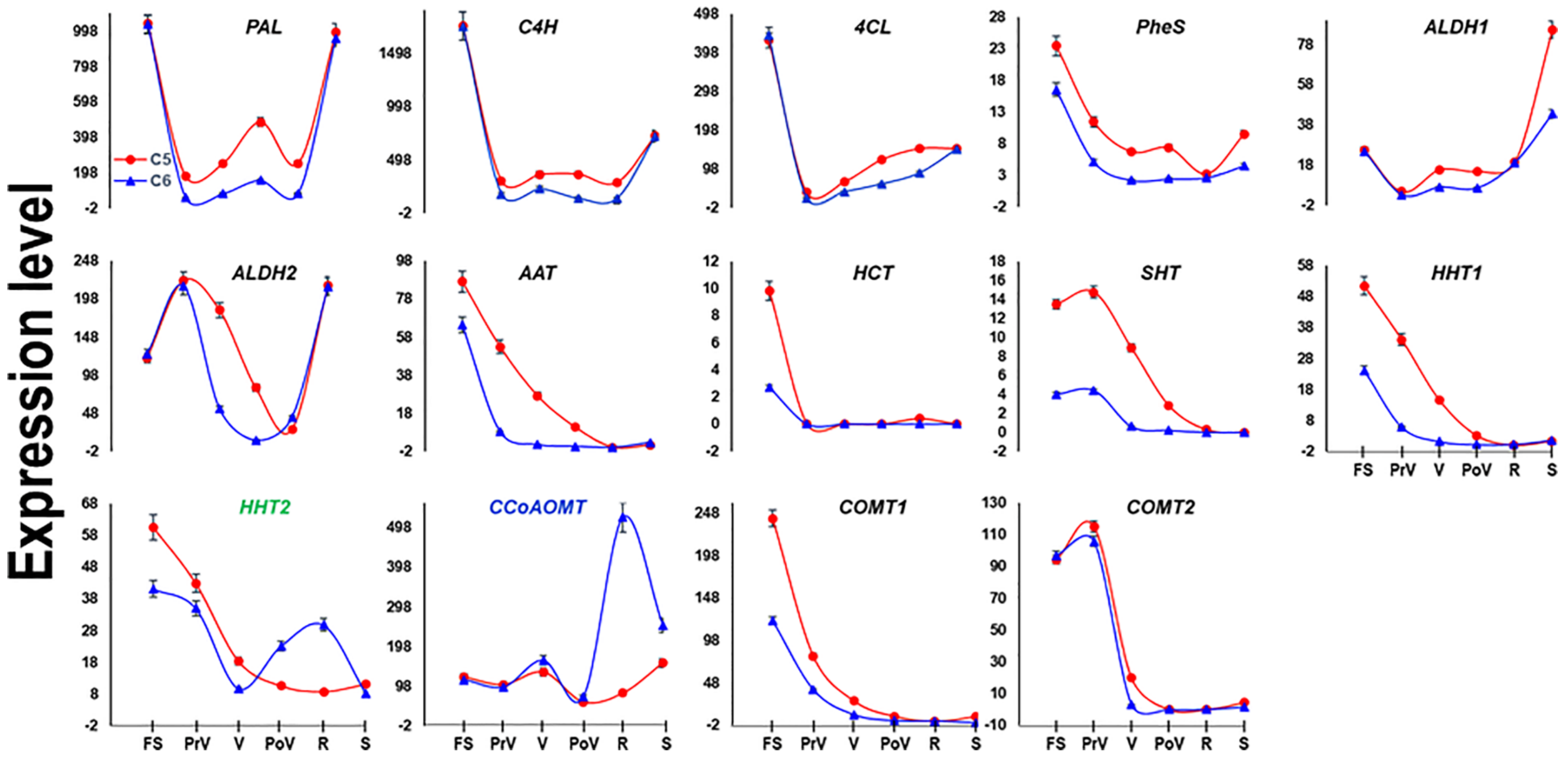

Fig. S20. Hydroxycinnamic Acids Pathway. The curves show the expression pattern of genes involved in hydroxycinnamic acids pathway that were assessed by qPCR at different berry developmental stages, including fruit-set (FS), pre-véraison (PrV), véraison (V), post-véraison (PoV), ripe skin/flesh (R), and ripe seeds (S). *PAL*, Phenylalanine ammonia-lyase; *C4H*, trans-4-coumarate biosynthesis; *4CL*, 4-coumaroyl:CoA-ligase; *PheS*, phenylalanine ligase; *ALDH*, aldehyde dehydrogenase; *AAT*, anthocyanidin 3-*O*-glucoside 6''-*O*-acyltransferase; *HCT*, shikimate *O*-hydroxycinnamoyl transferase; *SHT*, spermidine hydroxycinnamoyl transferase; *HHT*, omega-hydroxypalmitate *O*-feruloyl transferase; *CCoAOMT*, caffeoyl-CoA *O*-methyltransferase; *COMT*, caffeic acid 3-*O*-methyltransferase. Blue-colored gene names represent the transcripts exhibiting different pattern, where gene significantly expressed in C6 at specific stages. However, green-colored gene showed different pattern, where the gene was initially more abundant in C5, but latter the pattern was shifted at specific stages. Black-colored genes showed similar patterns with kinetics/levels between C5 and C6 genotypes.

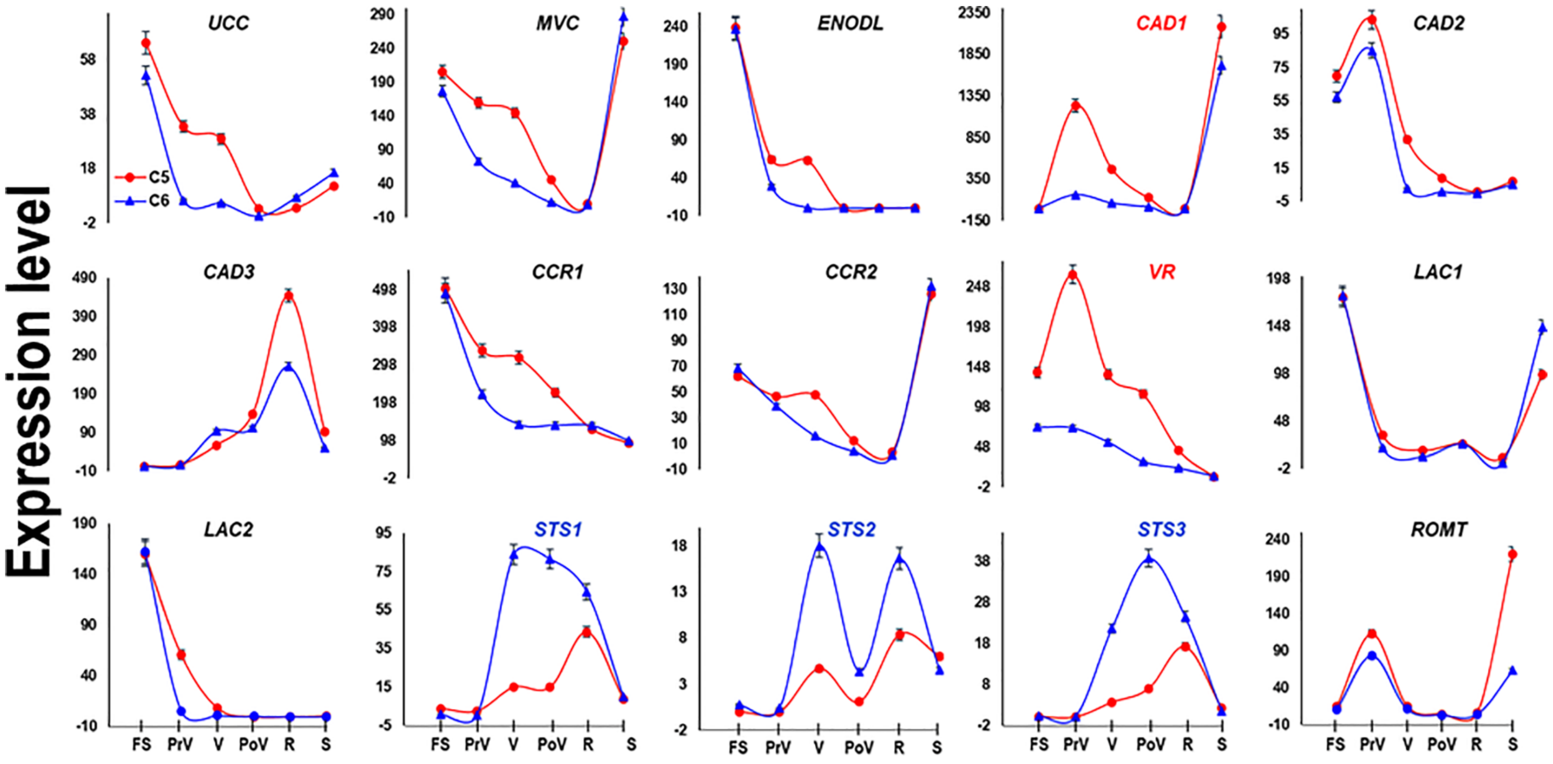

Fig. S21. Lignin and Pterostilbene Pathway. The curves show the expression pattern of genes involved in lignin and pterostilbene pathway that were assessed by qPCR at different berry developmental stages, including fruit-set (FS), pre-véraison (PrV), véraison (V), post-véraison (PoV), ripe skin/flesh (R), and ripe seeds (S). *UCC*, uclacyanin; *MVC*, mavicyanin; *ENODL*, early nodulin; *CAD*, cinnamyl alcohol dehydrogenase; *CCR*, cinnamoyl-CoA reductase; *VR*, vestitone reductase; *LAC*, laccase; *STS*, stilbene synthase; *ROMT*, trans-resveratrol di-*O*-methyltransferase. Red- and Blue-colored gene names represent the transcripts exhibiting different pattern, where gene significantly expressed in C5 or C6 at specific stages, respectively.

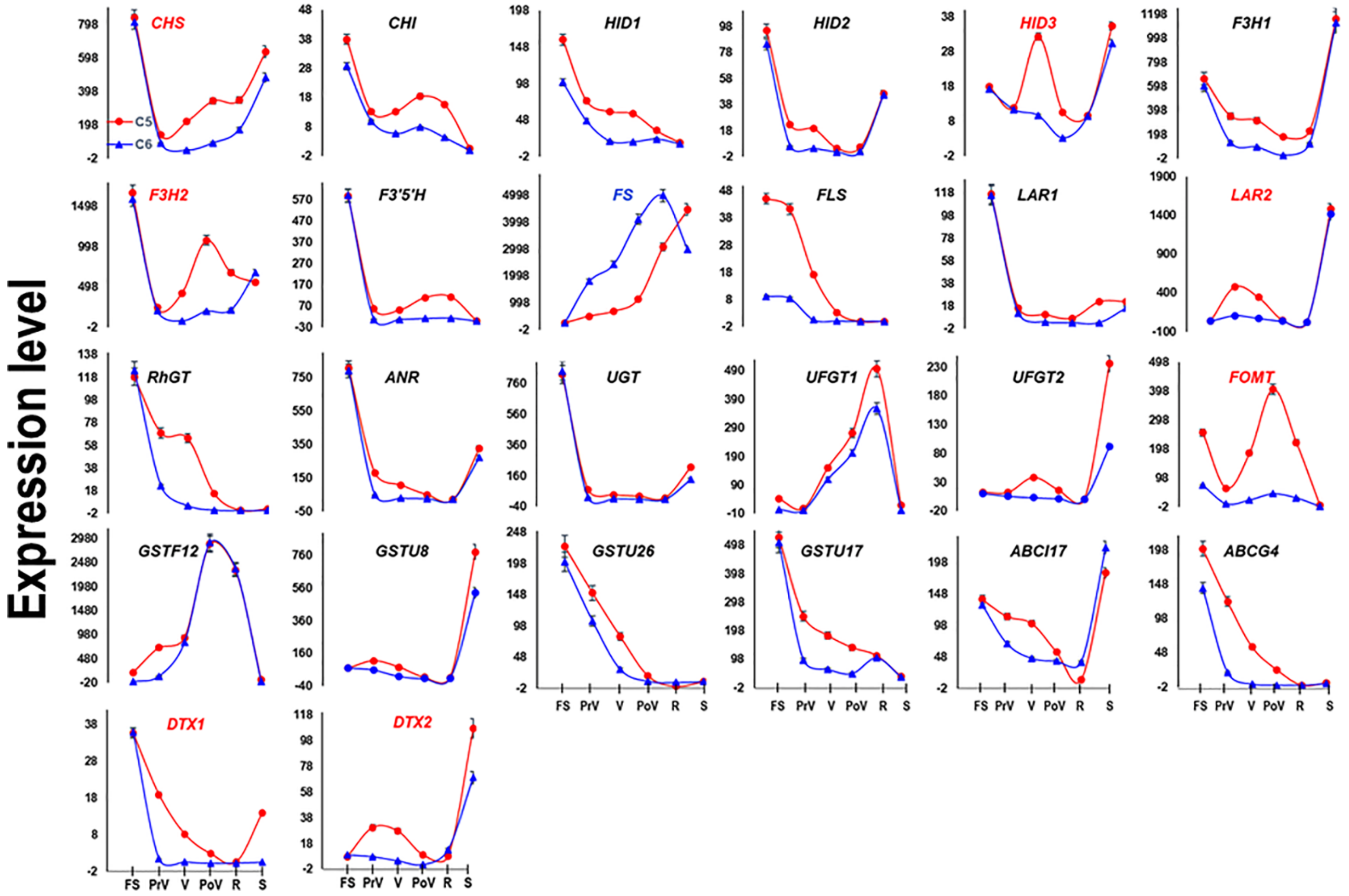

Fig. S22. Flavonoid Pathway. The curves show the expression pattern of genes associated in flavonoid pathway that were studied by qPCR at different berry developmental stages, including fruit-set (FS), pre-véraison (PrV), véraison (V), post-véraison (PoV), ripe skin/flesh (R), and ripe seeds (S). *CHS*, chalcone synthase; *CHI*, chalcone isomerase; *HID*, 2-hydroxyisoflavanone dehydratase; *F3H*, flavonoid 3'-monooxygenase; *F3'5'H*, flavonoid 3'5'-hydroxylase; *FS*, flavonol synthase; *FLS*, flavonol sulfotransferase; *LAR*, leucoanthocyanidin reductase; *RhGT*, anthocyanidin 5,3-*O*-glucosyltransferase; *ANR*, anthocyanidin reductase; *UGT*, gallate 1-beta-glucosyltransferase; *UFGT*, anthocyanidin 3-*O*-glucosyltransferase; *FOMT*, flavonoid 3,5-methyltransferase; *GST*, glutathione S-transferase; *ABC*, ABC transporter; *DTX*, protein detoxification. Red- and blue-colored gene

names represent the transcripts exhibiting different paterns, where gene significantly expressed in C5 and C6 at specific stages, respectively. Black-colored gene names represent the transcripts displaying similar pattens with different kintecs in C5 and C6 genotypes.

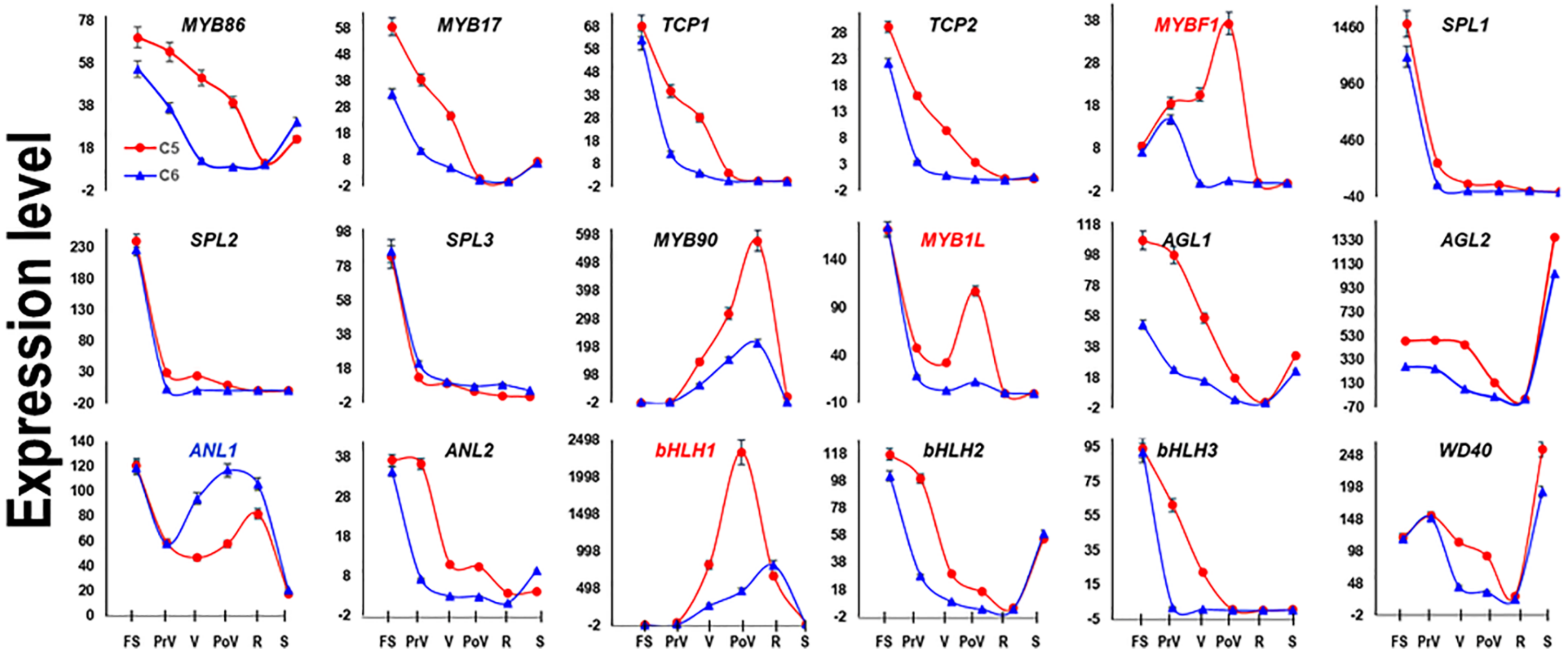

Fig. S23. Transcription factors regulating the phenylpropanoid/flavonoid pathway. The curves show the expression pattern of genes involved in the regulation of flavonoid and lignin pathways that were assessed by qPCR at different berry developmental stages, including fruit-set (FS), pre-véraison (PrV), véraison (V), post-véraison (PoV), ripe skin/flesh (R), and ripe seeds (S). *MYB*, myb-related regulatory gene; *TCP*, Teosinte branched1/Cincinnata/proliferating cell factor; *SPL*, squamosa promoter-binding-like protein; *AGL*, Agamous MADS-box protein; *ANL*, homeobox-leucine zipper protein anthocyaninless; *bHLH*, basic helix-loop-helix; *WD40*, tryptophan-aspartic acid repeat. Red- and blue-colored gene names represent the transcripts displaying different paterns, where gene significantly expressed in C5 and C6 at specific stages, respectively. Black-colored gene names represent the transcripts exhibiting similar pattens with different kintecs in C5 and C6 genotypes.

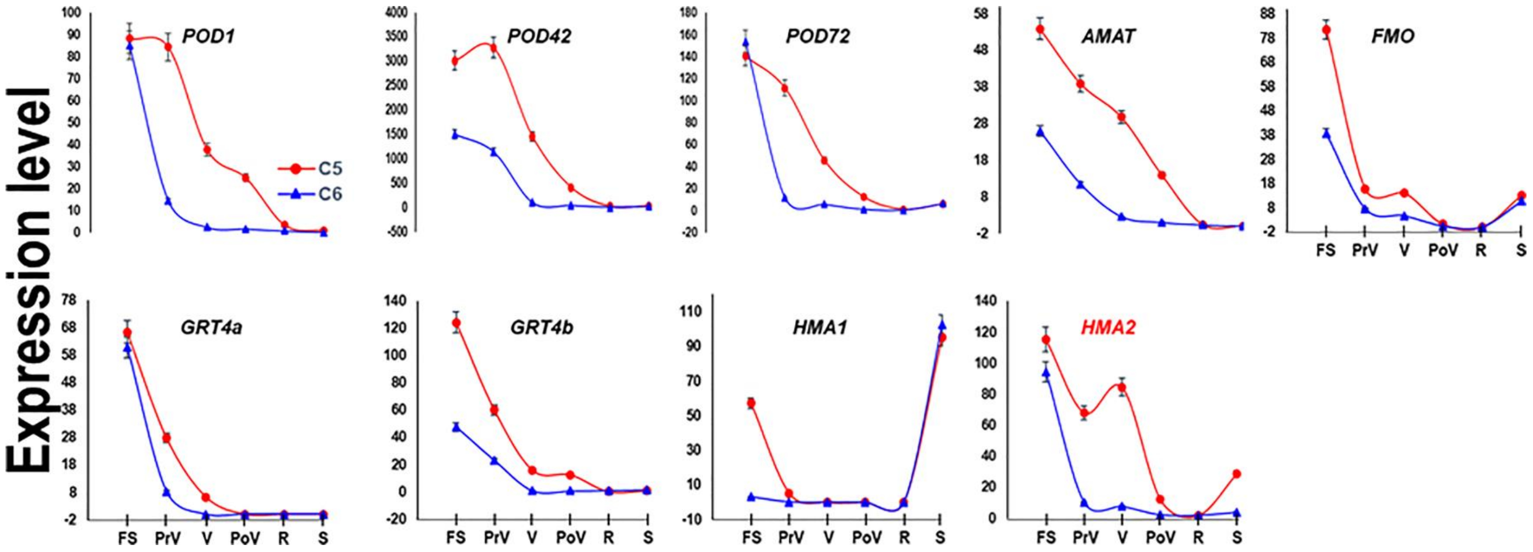

Fig. S24. Genes encoding proteins that are reported to contribute synergistically to the phenylpropanoid/flavonoid biosynthesis although they are not depicted its pathway. The curves show the expression pattern of genes, contributing to the phenylpropanoid/flavonoid that were assessed by qPCR at different berry developmental stages, including fruit-set (FS), pre-véraison (PrV), véraison (V), post-véraison (PoV), ripe skin/flesh (R), and ripe seeds (S). *POD*, peroxidase; *AMAT*, methanol *O*-anthraniloyltransferase; *FMO*, flavin-containing monooxygenase; *GRT4*, UDP-glycosyltransferase activity; *HMA*, heavy metal transport/detoxification superfamily protein.
